# High‐fidelity CRISPR/Cas12a dual‐crRNA screening reveals novel synergistic interactions in hepatocellular carcinoma

**DOI:** 10.1002/ctm2.1758

**Published:** 2024-07-28

**Authors:** Qian Chen, Minhui Pang, Peng Chen, Zihao Zhou, Jun Lei, Boxiao He, Zaiqiao Sun, Chonil Paek, Baowei Jing, Yankang Wu, Shiqi Liu, Yongshun Chen, Lei Yin

**Affiliations:** ^1^ State Key Laboratory of Virology Hubei Key Laboratory of Cell Homeostasis Department of Biochemistry and Molecular Biology College of Life Sciences Wuhan University Wuhan China; ^2^ Department of Case Statistics The Sixth Hospital of Wuhan Affiliated Hospital of Jianghan University Wuhan China; ^3^ Department of Clinical Oncology Renmin Hospital of Wuhan University Wuhan University Wuhan China

**Keywords:** CeCas12a, combinatorial screening of double knockouts, hepatocellular carcinoma

## Abstract

CRISPR/Cas12a‐based combinational screening has shown remarkable potential for identifying genetic interactions. Here, we describe an innovative method for combinational genetic screening with rapid construction of a dual‐CRISPR RNA (crRNA) library using gene splicing through overlap extension PCR (SOE PCR) and the adoption of CeCas12a, which we previously identified with strict PAM recognition and low off‐targeting to guarantee fidelity and efficiency. The custom‐pooled SOE crRNA array (SOCA) library for double‐knockout screening could be conveniently constructed in the laboratory for widespread use, and the CeCas12a‐mediated high‐fidelity screen displayed good performance even under a negative selection screen. By designing a SOCA dual‐crRNA library that covered most of the kinase and metabolism‐associated gene targets of FDA‐approved drugs implicated in hepatocellular carcinoma (HCC) tumourigenesis, novel cross‐talk between the two gene sets was negatively selected to inhibit HCC cell growth in vitro and in vivo and was validated using virtual double‐knockdown screening based on TCGA databases. Thus, this rapid, efficient and high‐fidelity double‐knockout screening system is promising for systemically identifying potential genetic interactions between multiple gene sets or combinations of FDA‐ approved drugs for clinical translational medicine in the future.

## Graphical abstract

1. High‐fidelity CRISPR/Cas12a dual‐crRNA screening identifies synergistic interactions of Raf1‐Pkm2 in the development of hepatocellular carcinoma.

2. Raf1 and Pkm2 display a significant clinical correlation in tissue microarray and closely correlated with HCC pathological grading.

3. Low expression of Raf1‐Pkm2 significantly improves patient survival in TCGA and GEO databases.

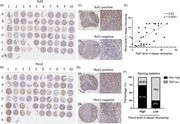

## 1 INTRODUCTION

CRISPR/Cas9‐based single‐gene library screening has been extensively used to explore drug targets and cancer therapy.^1^, ^2^, ^3^ However, many functional genes in organisms exert combinational effects under specific physiological conditions to drive abnormal life activities, such as cancer progression and immune escape, and traditional single‐gene library screening has been unable to evaluate genetic interactions systematically. Innovative strategies are therefore required. Combinational genetic screening or gene interaction mapping may be a better choice, as it is performed to study complicated genetic interactions systematically.^4^, ^5^, ^6^, ^7^, ^8^ Although the CRISPR/Cas9 system can mediate combinatorial genetic screening, it requires more sgRNA cassettes for double or multiple‐knockout library screening, increasing the complexity of library design, cloning and analysis. The off‐target effect caused by Cas9 can lead to significant differences in the number of disruptive mutations between sgRNAs targeting the same genes, which can lead to false‐positive screening results and ultimately affect the reliability of library screening.^9^, ^10^, ^11^, ^12^, ^13^, ^14^ These factors have limited the development of Cas9 for combinatorial genetic screening.

With the broad use of multigene knockout technology using CRISPR‐Cas12a, recent studies have begun to explore its potential in combinatorial genetic screening.^15^, ^16^, ^17^ The simple assembly of Cas12a (known as Cpf1), which belongs to the class 2 (type V) CRISPR system, has emerged as a versatile platform for multigene editing using a single crRNA array, making it convenient for double‐gene knockout screening.^18^, ^19^ The most notable feature of Cas12a, which is crucial for library screening, is that its off‐target rate is significantly lower than that of Cas9. However, redundant library design and the long synthesis cycle of oligo pools hinder the wide application of this technology. Additionally, unlike the single gene library screening for over 25 000. Human genes, the double‐knockout library screening could barely cover all combinations of human genes (above 6 × 10^8^ combinations), with the need for the barely possible amount of 3 × 10^11^ cells in one screening trial. Specialised multigene knockout library screening is one such option. Therefore, we developed a set of lab‐made construction methods for a specialised double‐knockout library. In this study, we designed long‐chain primers with complementary ends to amplify the library core fragments (DR‐spacer1‐DR‐complementary end‐DR‐spacer2‐DR) using gene splicing via overlap extension PCR (SOE PCR) and conducted in‐fusion cloning with an appropriate vector to generate an (SOE crRNA array) library. Unlike multiple crRNA arrays targeting gene combinations in traditional long oligo pools, the SOCA (custom‐pooled SOE crRNA array) library simplifies the crRNA array composition and construction. Further, simplified crRNAs generally lower the risk of off‐targeting and rapidly check the editing rate, and off‐targeting before screening can be easily conducted to guarantee efficiency and accuracy.

In this study, we identified a good Cas12a candidate, CeCas12a, a novel nuclease identified in our previous work, presenting high gene‐editing efficiency and low off‐target effects for double‐knockout library screening. A novel, rapid, lab‐made double‐knockout library, the SOCA library, was developed. The double‐knockout screening was designed to cover most of the kinase‐ and metabolism‐associated gene targets of FDA‐approved drugs implicated in hepatocellular carcinoma (HCC) tumourigenesis, both of which have been implicated in the malignant proliferation of HCC, and for the first time we systematically explored the interactions between these two gene sets. Novel interactions between kinases and metabolism‐associated genes affecting HCC growth have been identified and validated in vitro and in vivo. The virtual double‐knockdown screening was innovatively conducted to check the influence of all gene pair combinations on the HCC survival rate, aiming to verify the clinical translational potential of the identified gene pairs. Our data suggest that SOCA library screening using the novel CeCas12a with high efficacy and fidelity can mediate rapid, reliable and efficient double‐knockout screening. This double‐knockout screening of gene targets of FDA‐approved drugs reveals great clinical translational potential for discovering novel drug combinations for disease treatment.

## 2 RESULTS

### 2.1 The CeCas12a nuclease with high efficacy and fidelity for accurate library screening

CRISPR‐based library screening can be divided into positive selection screen and negative selection screen. In the positive selection screen, a certain selection pressure is applied to the cell library that has successfully integrated the sgRNA; as a result, only a few cells with the target phenotype survive to be enriched for key genes. The negative selection screen is the opposite, where the surviving cells do not have the targeted phenotype; therefore, sgRNA/crRNA abundance at different time points must be compared to identify differential sgRNA/crRNA to identify key genes. This requires that the crRNAs targeting the library genes have high activity to improve the efficiency and accuracy of the negative selection screen.

Guide RNAs with high‐efficiency scores and balanced (30–70%) GC content may be optimal for efficient genome editing.^20^, ^21^ Based on these principles, we designed predictive crRNAs for all candidate genes in the SOCA library, which covers most kinase genes and metabolism‐associated genes derived from molecular targets of FDA‐approved drugs involved in HCC tumourigenesis. To verify the accuracy of our predicted crRNA activity, we determined the crRNA activity for 27 library candidate genes in Lb2Cas12a‐positive cells, and all crRNAs showed significant cleavage activities (Figure S1A).

**FIGURE 1:**
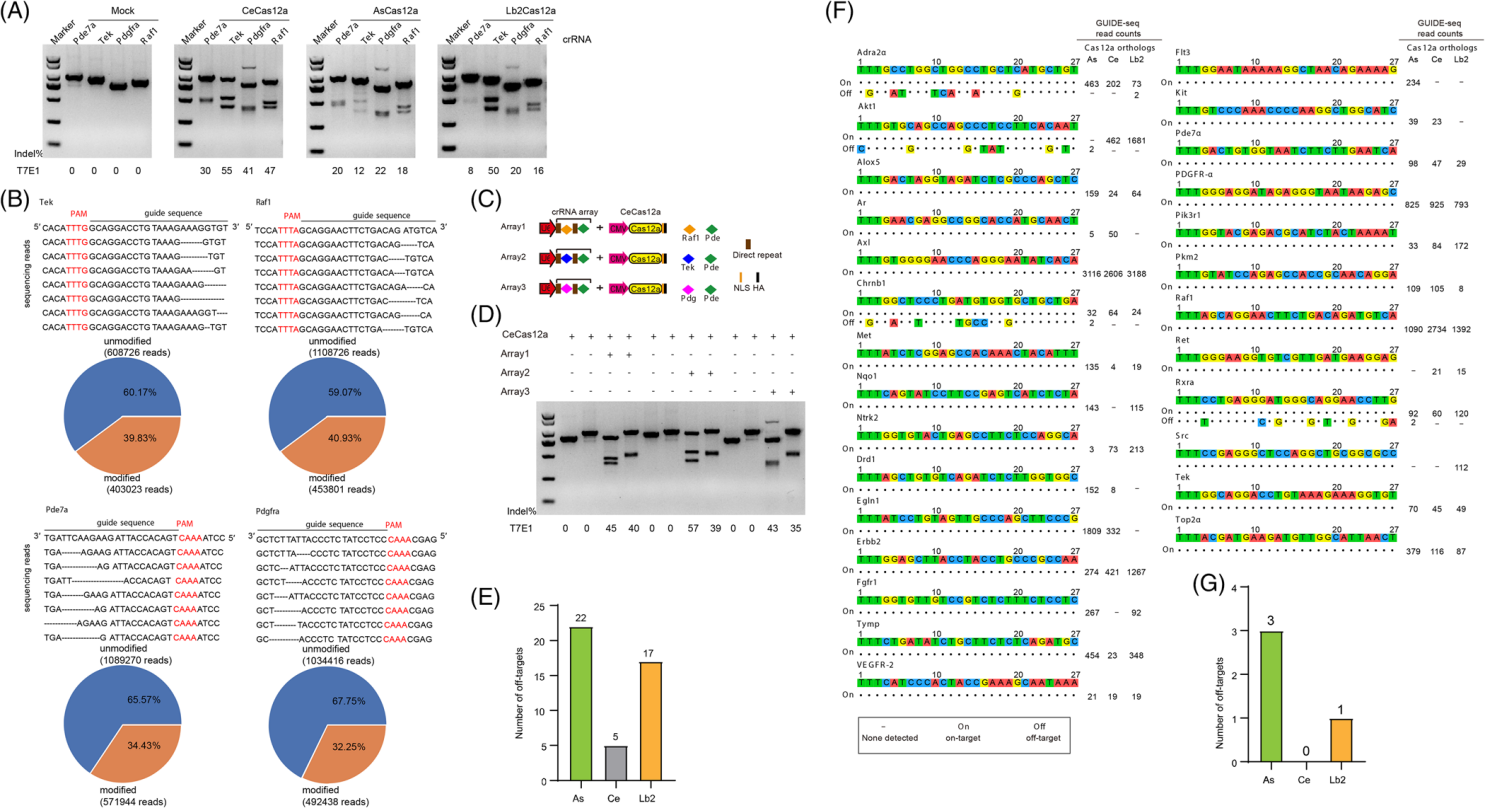
**The CeCas12a nuclease with high efficacy and fidelity for accurate library screening. (A)** Activities of CeCas12a, AsCas12a, and Lb2Cas12a on targets with TTTV PAM sequences across four endogenous targets measured using T7 endonuclease I assay. **(B)** Sequencing reads show mutations of CeCas12a‐mediated gene editing in the Tek, Raf1, Pde7a and Pdgfra loci (Top). Dashes represent the DNA deletions. Indel frequencies for four endogenous target sites were measured using deep sequencing (Bottom). **(C)** Schematic of double‐knockout CRISPR array constructs with the U6 direct repeat spacer cassette containing Raf1 and Pde7a, Tek and Pde7a, Pdgfra and Pde7a protospacer sequences. **(D)** Indel frequencies were analysed using the T7E1 assay after transduction with the CRISPR array and CeCas12a lentivirus. **(E)** Summary of the total number of off‐target sites identified using GUIDE‐ seq for AsCas12a, CeCas12a, and Lb2Cas12a with crRNAs targeting 3 endogenous sites in 293T. **(F)** Genome‐wide specificities of Cas12a orthologues match crRNAs targeting all candidate genes in the SOCA library. On‐target and off‐target sites for AsCas12a, CeCas12a and Lb2Cas12a with crRNAs targeting 27 candidate genes were determined using GUIDE‐seq in Hepa1‐6 cells. **(G)** Summary of the total number of off‐target sites identified using GUIDE‐seq for AsCas12a, CeCas12a and Lb2Cas12a with crRNAs targeting 27 candidate genes.

Identifying Cas12a orthologues with high editing activity and low off‐target rates for library candidates is crucial for negative screening. CeCas12a, a novel nuclease identified in our previous study, showed stringent PAM recognition to achieve lower off‐target editing rates than AsCas12a, LbCas12a and enAsCas12aHF1, with high editing activity for genome editing.^22^, ^23^ In this study, we systematically evaluated the performance of CeCas12a and two canonical Cas12a orthologues, AsCas12a and Lb2Cas12a, on SOCA library candidates. The performances of CeCas12a, AsCas12a and Lb2Cas12a were checked against several endogenous targets in the library candidates through T7E1 assays and against all library candidate targets through the genome‐wide unbiased identification of double‐stranded breaks enables by sequencing (GUIDE‐seq). CeCas12a showed high editing activity in Hepa1‐6 cells (Figure 1A and S2A). The gene editing (insertion or deletion) efficiencies of CeCas12a were consistent with T7E1 assay results (Figure 1B and S3A–C). To explore whether CeCas12a is capable of editing multiple targets with a CRISPR array in Hepa1‐6 cells, we designed a variety of arrays from library candidates and demonstrated that CeCas12a showed excellent editing activity for double targets using independently replicated T7E1 assays (Figure 1C and D and S2B). To systematically evaluate the specificity of CeCas12a, AsCas12a and Lb2Cas12a, the GUIDE‐seq was adopted to check genome‐wide on‐ and off‐target sites in human 293T and mouse Hepa1‐6 cells. The results of the GUIDE‐seq revealed that CeCas12a had the best specificity among the three tested Cas12a orthologues to maintain high fidelity for the whole target sites of our library (Figure 1E‒G and S4). Taken together, our data suggest that CeCas12a could be a good choice in performing negative selection screen using the SOCA library.

### 2.2 CeCas12a‐based SOCA library performed double‐knockout screening through efficient negative selection

**FIGURE 2:**
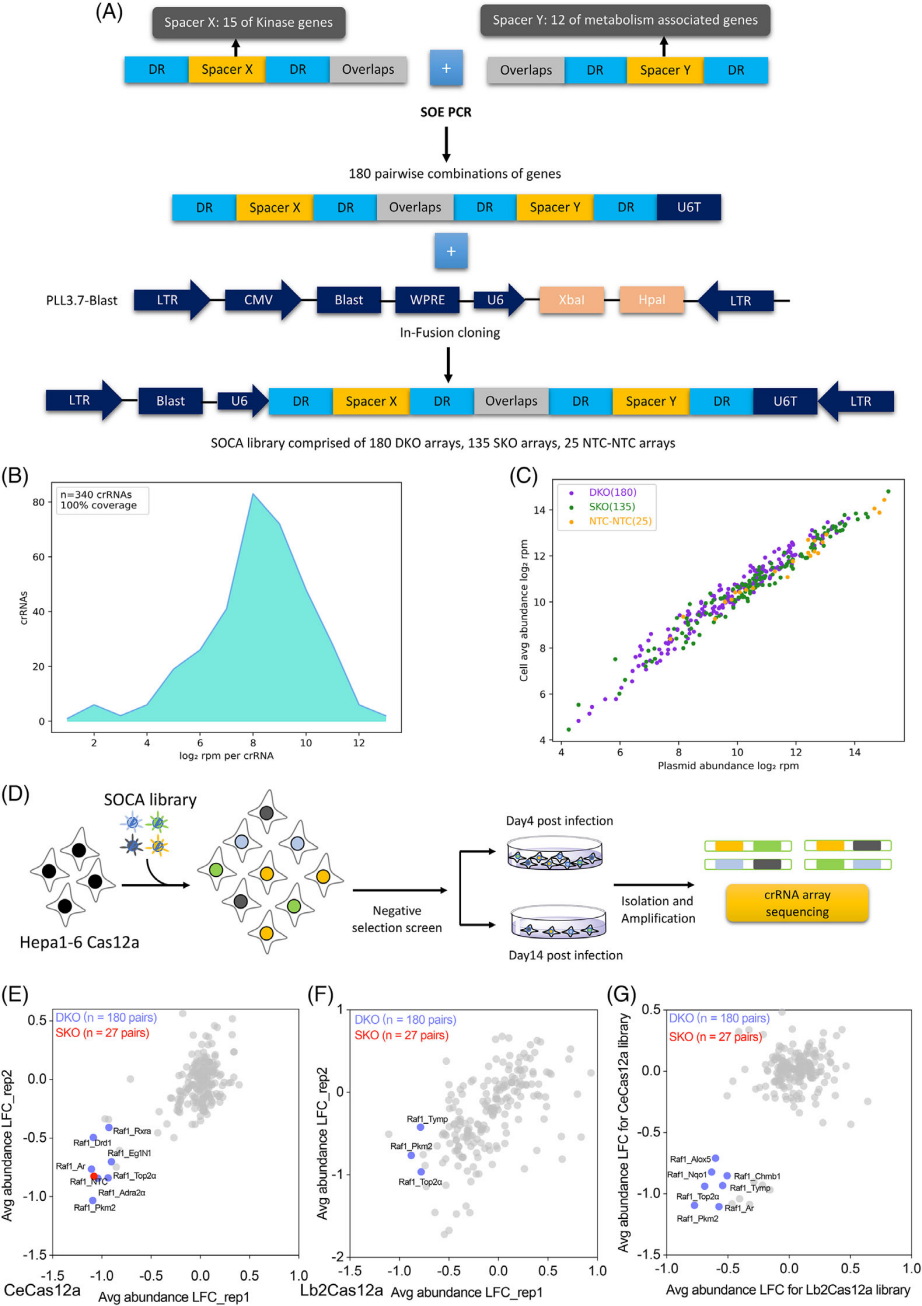
**CeCas12a‐based SOCA library performed double‐knockout screening through efficient negative selection. (A)** Experimental workflow for constructing the SOCA library. Guide‐seq and T7E1 assays determined the optimal Cas12a nuclease and crRNA for 27 candidate genes in the SOCA library and designed overlapping complementary primers to amplify library core fragments using SOE PCR. The core fragments containing 340 combinations were cloned into PLL3.7‐blast vector using in‐fusion cloning. **(B)** A density plot depicting the abundance distribution of the SOCA library. NGS sequencing determined 100% library coverage, consisting of 340 crRNA arrays. **(C)** Scatter plot depicting SOCA library abundance in plasmids (*n* = 1) and averaged cell pools (*n* = 3). DKO: double knockout; SKO: single knockout. **(D)** Schematic of SOCA library‐mediated combinatorial screening in vitro. **(E)** The scatter plot compares the LFC of crRNA abundance on two independent replicate screenings in CeCas12a‐positive cells. LFC: log fold‐change; the value was used to represent the ratio of the final crRNA rpm (Day 14) to the initial crRNA rpm (Day 4); DKO: double knockout; SKO: single knockout; blue dots: DKO combination identified in SOCA library screening; red dots: SKO combination identified in SOCA library screening. **(F)** The scatter plot compares the LFC of crRNA abundance on two independent replicate screenings in Lb2Cas12a‐positive cells. **(G)** The scatter plot compares SOCA library screening results in CeCas12a‐ and Lb2Cas12a‐positive cells.

Multiple crRNAs were designed for each candidate gene and the optimal crRNA was selected for each of the 27 candidate genes of kinases and metabolism‐associated genes involved in hepatocellular carcinoma (HCC) tumourigenesis. Using SOE PCR (gene splicing via overlap extension PCR) technology, we spliced two crRNA spacers with complementary ends to synthesise a SOCA library containing 340 crRNAs (180 DKO arrays, 135 SKO arrays and 25 NTC‐NTC arrays) (Figure 2A). Deep sequencing revealed a 100% coverage of the SOCA library (Figure 2B). In addition, we were able to observe a strong correlation between the cell pool and the SOCA library plasmid using sequencing the average abundance log_2_ rpm (reads per million) of crRNAs in the plasmids and cells (Figure 2C).

To identify synergistic combinations of kinases and metabolic genes in the SOCA library, we performed a negative selection screen on CeCas12a/Lb2Cas12a‐positive Hepa1‐6 cells and determined the variation in crRNA abundance from 4 to 14 days. (Figure 2D). To analyse the variation in crRNA abundance in target cells, we calibrated each crRNA array read count to reads per million (RPM) and used the fold‐change (FC) value to represent the ratio of the final crRNA RPM (Day 14) to the initial crRNA RPM (Day 4), followed by converting the FC value to a log_2_ FC (LFC) value to analyse the variation in crRNA abundance. After 14 days of negative selection screen, we found that the results of the double‐knockout screening on CeCas12a/Lb2Cas12a‐positive Hepa1‐6 cells correlated with the corresponding biological replicates (Figure 2E and F), and CeCas12a screening was correlated with Lb2Cas12a screening (Figure 2G). Furthermore, through the negative selection screen, we identified several novel knockout combinations for kinases and metabolism‐associated genes implicated in HCC tumourigenesis, such as Raf1_Pkm2 and Raf1_Top2α, Raf1_Alox5, Raf1_Drd1, Raf1_Nqo1 and Raf1_Rxra, which belong to the Raf1‐associated gene pairs, and showed that hepa1‐6 cells are sensitive to Raf1 perturbation. This result is consistent with the earliest Cas12a crRNA library screen performed by Chow et al.,^15^ where most gene pairs had stationary crRNAs that may play a dominant role.

To assess whether overlapping complementary fragments (overlaps) in the core fragments of the SOCA library could affect the accuracy of SOCA library screening, we constructed all double‐knockout plasmids (180 dual‐crRNA arrays without overlaps) derived from the SOCA library (Figure S5A). We integrated 180 double‐knockout plasmids as a parallel library and performed parallel combinatorial screening in vitro (Figure S5B). We confirmed that the coverage of the parallel libraries was 100%, and the library homogeneity was approximately 11 (Figure S5C). In addition, we compared the distribution of SOCA libraries and parallel libraries in the plasmid and cell pools by scatter plot, and observed that the distribution of crRNA abundance of parallel libraries in cells and plasmids was highly correlated and correlated with the abundance of SOCA libraries (Figure S5D). We used the parallel library to perform negative selection screening under the same screening conditions as the SOCA library and found that the identification results of the SOCA library and the parallel library were significantly correlated between the two Cas12a nucleases, with the number of combinations screened in the CeCas12a nuclease were significantly higher than that in the Lb2Cas12a nuclease, indicating the high fidelity potential of the CeCas12a nuclease (Figure S5E and F). The combinations identified in the SOCA library and the parallel library were all Raf1‐related gene pairs, including Raf1_Top2α, Raf1_Pkm2, Raf1_Adra, etc. Our data showed that overlap did not affect the accuracy of SOCA library screening.

### 2.3 In vivo combinatorial screening identified novel gene pairs implicated in HCC tumourigenesis

In vitro, screening at the cell line level has not been able to provide accurate feedback on the true phenotype of tumours because tumours are heterogeneous organs that are highly integrated into the organism rather than as separate entities.^24^ Therefore, in vivo screening is required to more accurately reflect the phenotype or intrinsic gene cluster variation in tumour and improve the translational potential of screening results.

**FIGURE 3:**
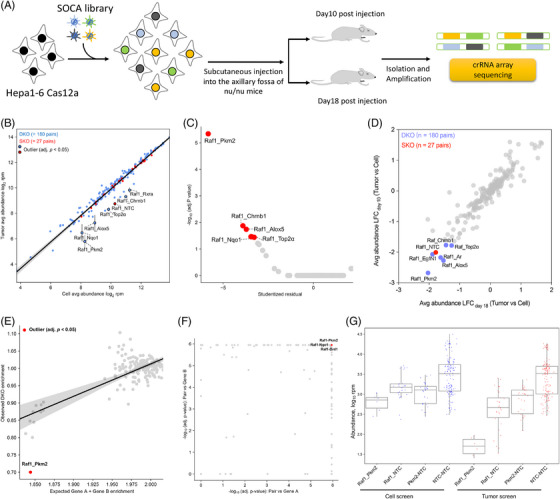
**In vivo combinatorial screening identified novel gene pairs implicated in HCC tumourigenesis. (A)** Schematic of SOCA library‐mediated combinatorial screening in vivo. **(B)** Scatter plot of SOCA library‐based combinational screening in cell pools (*n* = 3 cell replicates) versus primary tumours (*n* = 6 mice). The SOCA library is shown in a linear regression model (shaded areas indicate 95% CI). Significant outliers are enlarged (two‐sided outlier test, Benjamini–Hochberg‐adjusted *p* < .05). SKO: single knockout; DKO: double knockout. **(C)** The scatterplot compares the *p*‐value Benjamini–Hochberg adjusted from the outlier test to studentised residuals from linear regression analysis. **(D)** The scatter plot compares the LFC of tumour crRNA abundance at different time points (*r* = 0.93, Pearson's correlation). LFC: log fold‐change was used to represent the ratio of the final tumour crRNA rpm (Day 10 or Day 18) to the initial cell crRNA rpm (Day 4). **(E)** Comparison of observed DKO abundance enrichment with expected DKO abundance using NTC‐NTC normalisation. Significant outliers are coloured red (two‐sided outlier test, Benjamini–Hochberg‐adjusted *p* < .05). **(F)** Scatter plot depicting ‐log10 adjusted *p*‐values for each gene pair comparing DKO abundance to corresponding SKO abundance using a two‐sided Wilcoxon rank‐sum test. **(G)** Tukey's box plots depicting DKO, SKO, and NTC‐NTC abundance for Raf1‐Pkm2 under cell and tumour screens.

To identify specific perturbations that affect tumour growth in vivo, we transduced the SOCA library into CeCas12a‐positive Hepa1‐6 cells and added 10 µg/mL blasticidin to conduct selection until there were sufficient cells for injection. Subsequently, the cells were injected into nu/nu mice to assess variations in crRNA abundance in the primary tumour (Figure 3A). Previous studies have found that linear regression models of crRNA abundance between cell lines and tumours can identify specific combinations.^15^ Here, we took the average of the crRNA arrays for each SKO and used linear regression models to show the variation trend in crRNA abundance for all corresponding gene pairs in cell pools and tumours. In linear regression models, we identified Raf1‐associated gene pairs in primary tumours relative to cell pools including Raf1‐Pkm2, Raf1‐Top2α, Raf1‐Nqo1, Raf1‐Alox5, Raf1‐Chrnb1 and Raf1‐ Rxra in the model (outlier test, adjusted *p* < .05) (Figure 3B). This is consistent with the majority of gene pairs derived from the cell pool‐screening model. Furthermore, we analysed the adjusted *p*‐value compared to the studentised residuals from the linear regression and identified the most significant gene pair: Raf1‐Pkm2 (Figure 3C). By analysing the crRNA abundance variation in cell pools and tumours, we used the log fold‐change (LFC) to present the ratio of tumour crRNA abundance at different time points to crRNA abundance in the cell pool, thus exploring the abundance variation of tumours relative to the initial cell pool at different periods (Figure S6A and B). We observed that the LFC of the tumours on days 10 and 18 was significantly correlated. Moreover, the identified gene pairs were strongly consistent with the outliers from the linear regression (Figure 3D). To verify whether the identified combinations have synergistic effects, specific mathematical models are required. Here, we used two well‐established mathematical models to validate the identified gene pairs. First, we calculated significant outliers in the linear regression model based on the expected DKO versus the observed DKO under NTC‐NTC normalisation, and Raf1‐Pkm2 was identified as the only significant outlier (Figure 3E). Second, we analysed the combinational effect of gene pairs based on pairs versus corresponding single genes using the two‐sided Wilcoxon rank‐sum test and observed that the Raf1‐Pkm2, Raf1‐Nqo1, and Raf1‐Drd1 gene pairs were statistically significant compared to their corresponding single‐knockout gene pairs (Figure 3F). Furthermore, we analysed the variation in the DKO, SKO, and NTC‐NTC abundance of the Raf1‐Pkm2 gene pair in cell line and tumour screening model and observed that the DKO abundance of the Raf‐Pkm2 gene pair significantly decreased in the tumour screening model, whereas it remained almost unchanged in the single knockout and control groups (Figure 3G). These data show that there is a significant synergistic interaction between Raf1 and Pkm2 genes.

### 2.4 Screening of the SOCA library in human HCC cells confirmed the reproducibility of the identified gene pairs

**FIGURE 4:**
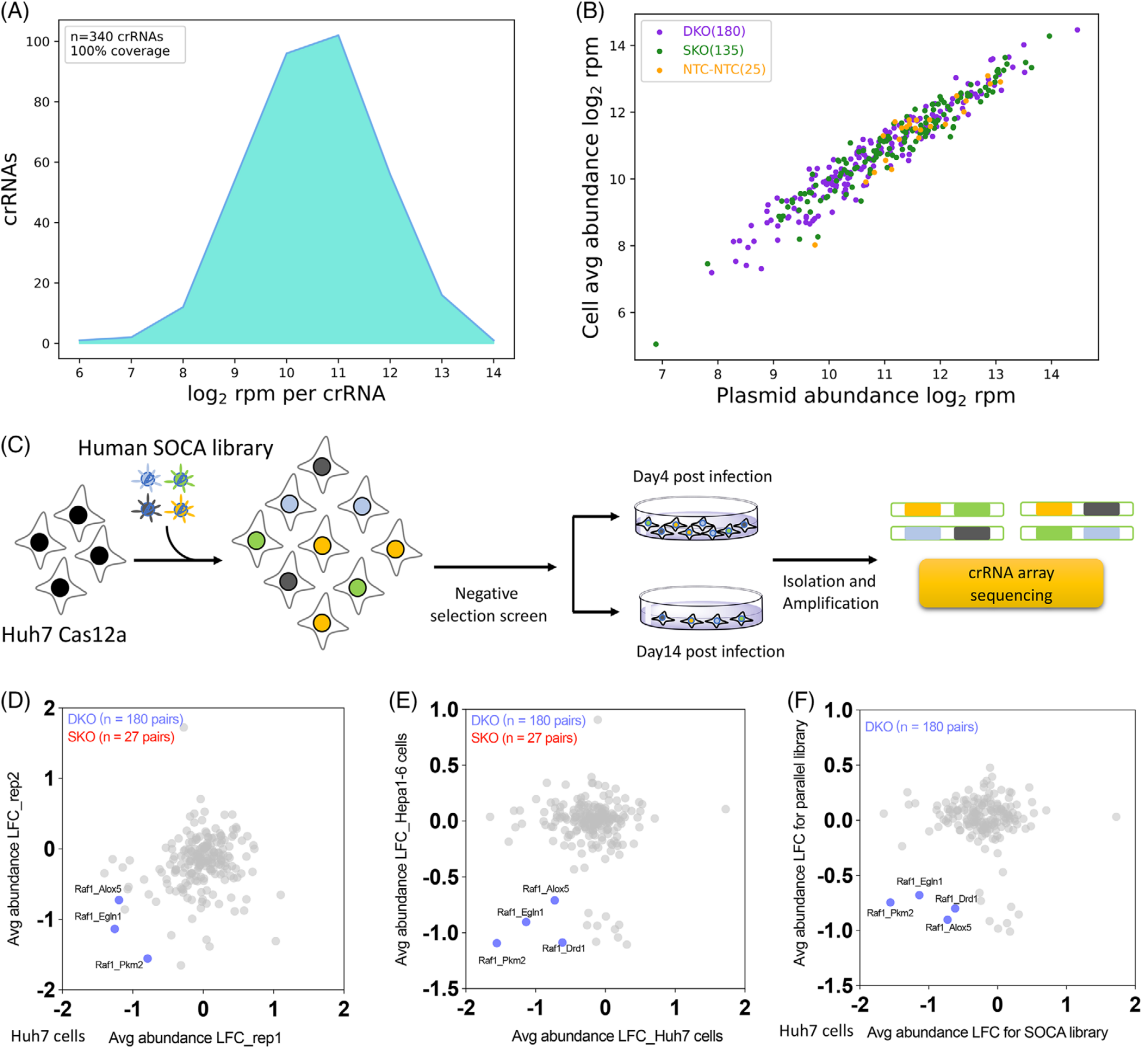
**Screening of the SOCA library in human HCC cells confirmed the reproducibility of the identified gene pairs**. (A) Density plot depicting the abundance distribution of human SOCA library. NGS sequencing determined 100% coverage of the library. (B) Scatter plot showing SOCA library abundance in plasmid (*n* = 1) and averaged cell pools (*n* = 3). (C) Schematic of in vitro combinatorial screening mediated by SOCA libraries. (D) Scatter plot compares the LFC of crRNA abundance on two independent replicate screens in CeCas12a‐positive cells. (D) Scatter plot compares that SOCA library screening results in Huh7 and Hepa1‐6 cells. (E) Scatter plot compares the screening results for Parallel library and SOCA library in CeCas12a‐positive cells.

Hepatocellular carcinoma is generally highly heterogeneous, as evidenced by the fact that the phenotypes and biological functions of HCC cell lines of different origins can differ markedly, and to validate that the combinations of our SOCA library identified in the Hepa1‐6 cells and mouse tumours could be reproduced in other HCC models, we selected the Huh7 cells (a human HCC cell line) and constructed Cas12a stably transduced Huh7 cell line and a human‐derived SOCA library, and NGS sequencing showed 100% library coverage and a significant correlation between SOCA library plasmids and cell pool (Figure 4A and B). We then used the human SOCA library lentiviruses to infect Huh7 cells and performed a 14‐day negative screen (Figure 4C). By comparing the log2 FC (LFC) values at Day 4 and Day 14, we found that the double‐knockout screening mediated by the SOCA library in Huh7 cells identified three combinations including Raf1‐Pkm2, Raf1‐Egln1 and Raf1‐Alox5 and Raf1‐Pkm2 was also identified in Huh7 cells and Hepa1‐6 cells (Figure 4D and E). Furthermore, by comparing the results with those of the parallel library screen, we found that all of the above Raf1‐related combinations were also identified (Figure 4F). These results indicate that the combinations identified in Hepa1‐6 cells can also be reproduced in other HCC models, further suggesting that Raf1‐associated combinations, especially Raf1‐Pkm2, play a stable biological function in HCC development.

### 2.5 In vitro and in vivo assays showed good therapeutic potential for Raf1‐Pkm2

**FIGURE 5:**
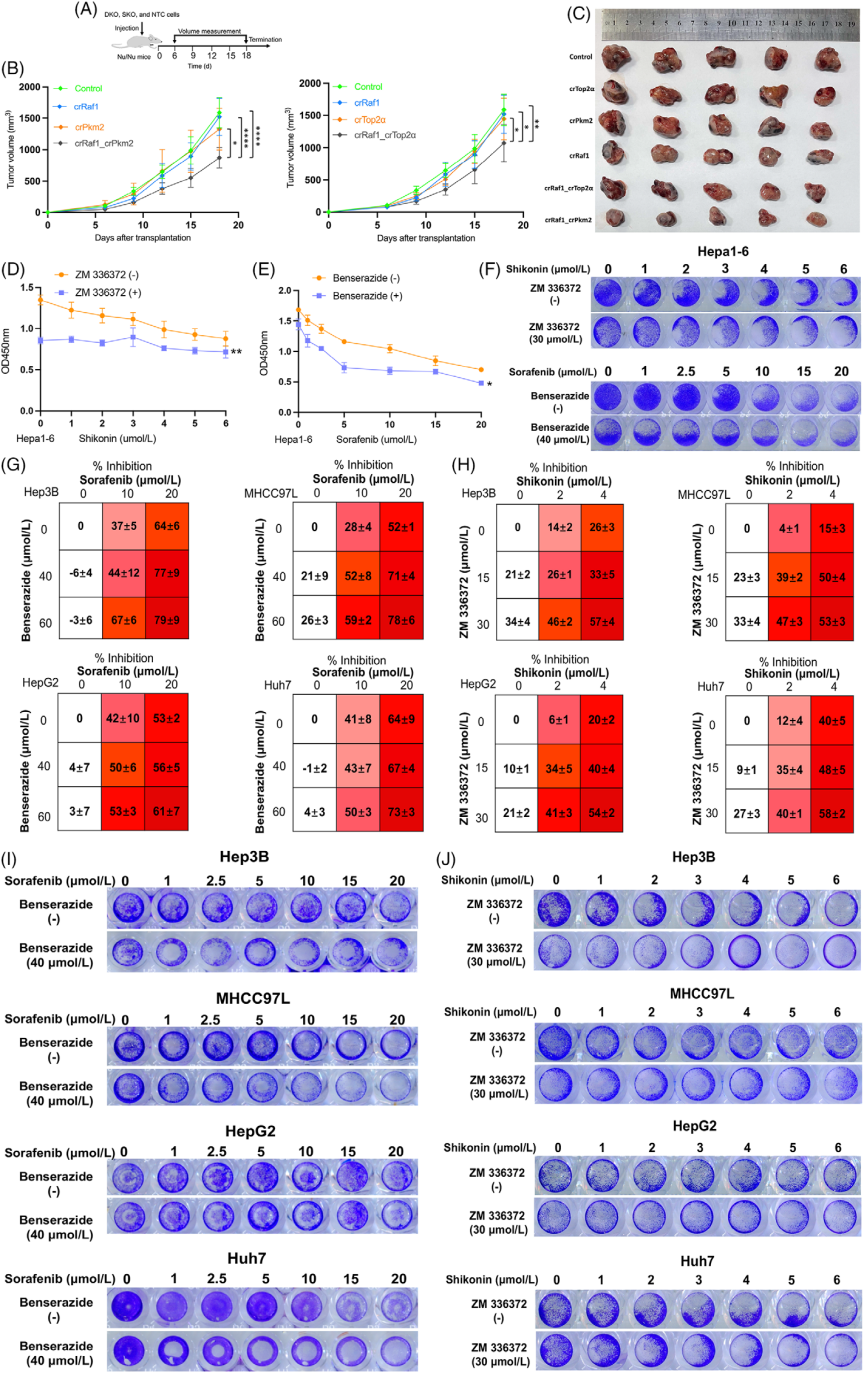
**In vitro and in vivo assays showed good therapeutic potential for Raf1‐Pkm2. (A)** A protocol to evaluate the growth of DKO, SKO and NTC cells of Raf1‐Pkm2 and Raf1‐Top2α in *nu/nu* mice. **(B)** Tumour volume growth curves in *nu/nu* mice transplanted with Control, crRaf1, crPkm2, crTop2α, crRaf1_crPkm2 and crRaf1_crTop2α cells. A two‐way ANOVA was used to evaluate the statistical significance. *p < .05, **p < .01, ****p < .0001. **(C)** Representative images of primary tumours harvested from nu/nu mice 18 days after transplanting with control, crRaf1, crTop2α, crRaf1_crTop2α and crRaf1_crPkm2 cells. **(D)** Quantitative analysis of ZM336372 in combination with shikonin after treatment of Hepa1‐6 cells in vitro. **(E)** Quantitative analysis of benserazide in combination with sorafenib after treatment of Hepa1‐6 cells in vitro. **(F)** Sorafenib in combination with benserazide and Shikonin in combination with ZM 336372 inhibited the proliferation of Hepa1‐6 cells in vitro. **(G)** Cell viability of sorafenib and benserazide combination treatment in Hep3B, MHCC97L, HepG2 and Huh7 cells. The CKK‐8 assay was used to measure cell viability. **(H)** Cell viability of shikonin and ZM 336372 combination treatment in Hep3B, MHCC97L, HepG2 and Huh7 cells. **(I)** Sorafenib in combination with benserazide inhibited the proliferation of Hep3B, MHCC97L, HepG2 and Huh7 cells. **(J)** Shikonin in combination with ZM 336372 inhibited the proliferation of Hep3B, MHCC97L, HepG2 and Huh7 cells.

Two gene pairs, Raf1_Pkm2 and Raf1_Top2α, were selected to check the inhibitory effect. We transplanted DKO, SKO and NTC cells of Raf1‐Pkm2 and Raf1‐Top2α into nu/nu mice (*n* = 5‒6) to measure tumour growth. The results showed that Raf1_Top2α and Raf1‐Pkm2 significantly inhibited tumour growth compared with the corresponding single‐knockout group and WT group (Figure 5A–C). Because both Raf1 and Pkm2 are FDA‐approved drug targets, we treated hepa1‐6 cells with sorafenib and benserazide, FDA‐approved drugs targeting Raf1 and Pkm2, respectively. The results showed that sorafenib combined with benserazide significantly inhibited the viability of Hepa1‐6 cells (Figure 5E and F). In addition, we validated the combined efficacy of FDA‐approved drugs in four human HCC cell lines and showed that sorafenib combined with benserazide may synergistically inhibit HCC cell proliferation. MHCC97L cells appeared to be the most sensitive to combination therapy (Figure 5G and I and S7D). Sorafenib is a multikinase inhibitor that simultaneously targets multiple genes, which may interfere with results. Therefore, we used specific compounds of Raf1 and Pkm2 to validate the synergistic effect between Raf1 and Pkm2 more accurately. Our results showed significant synergistic therapeutic effects of shikonin (an inhibitor of Pkm2) and ZM 336372 (an inhibitor of Raf1) in Hepa1‐6 cells and the four HCC cell lines (Figure 5D, F, H and J and S7E). In addition, we examined the therapeutic effects of GW5074 (another Raf1‐specific inhibitor) in combination with shikonin in various HCC cells. The CCK8 and clone formation assays indicated that GW5074 and shikonin exerted significant synergistic inhibitory effects (Figure S7A–C). Overall, the genetic and pharmacological inhibition models showed that Raf1 and Pkm2 play synergistic roles in the growth of HCC.

### 2.6 Virtual double‐knockdown screening based on TCGA databases confirmed favourable clinical efficacy for Raf1‐Pkm2

**FIGURE 6:**
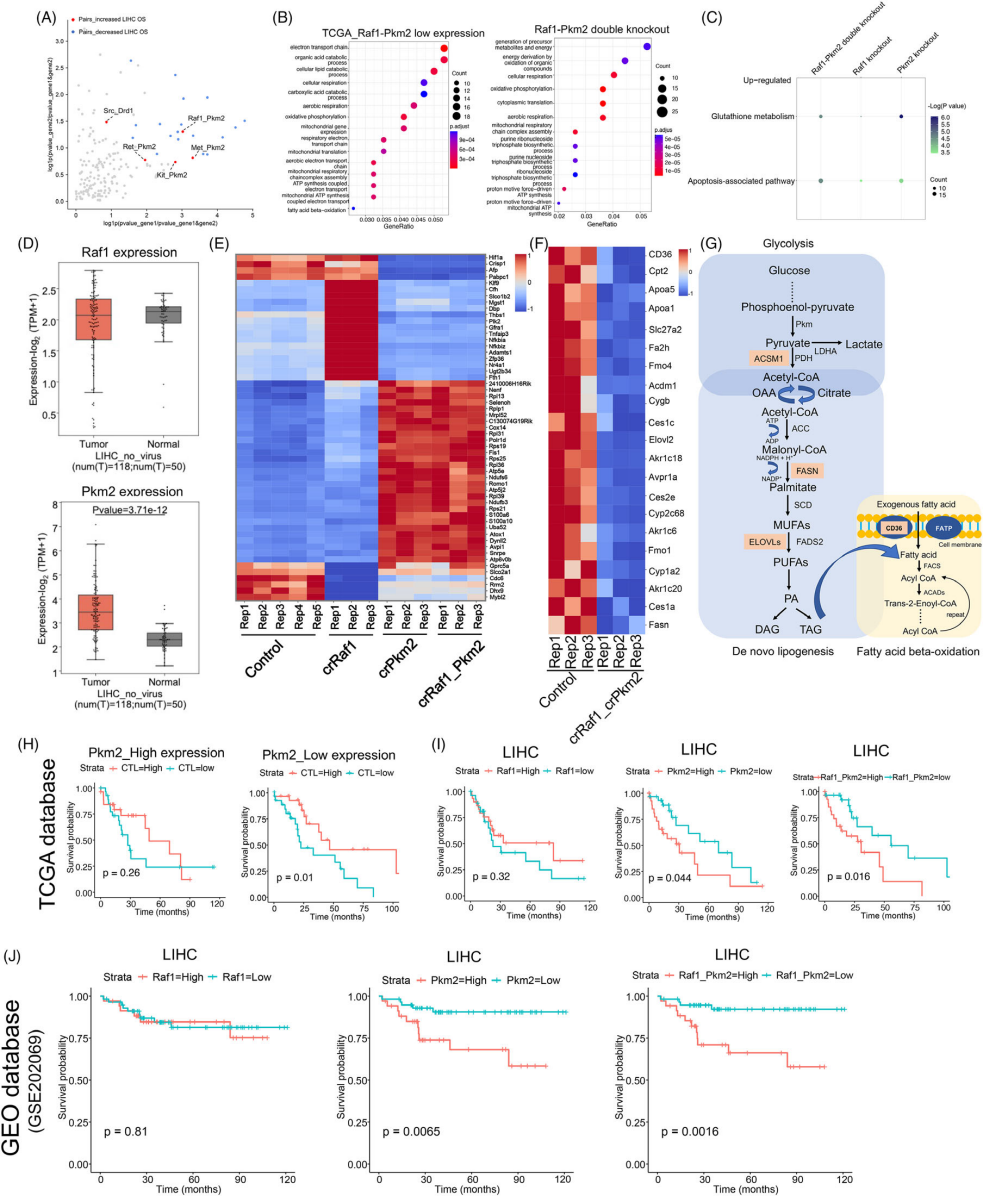
**Virtual double knockdown screening based on TCGA databases confirmed favourable clinical efficacy for Raf1‐Pkm2. (A)** Scatter plots show the correlation of overall patient survival from the HCC cohort with all double‐knockout combinations from the SOCA library. **(B)** The top 15 enriched pathways correlated with Raf1‐Pkm2 low expression or double knockout, as identified using GO analysis . **(C)** Representative KEGG pathways analysis showed that DEGs were enriched in gene sets associated with regulating cell apoptosis in crRaf1_crPkm2, crRaf1 and crPkm2 lentivirus‐transduced Hepa1‐6 cells. Dot colours were used to represent statistical significance, and dot size was used to represent the number of genes in the corresponding KEGG pathways. **(D)** Boxplots of Raf1 and Pkm2 expression in RNA‐seq samples from the TCGA database; *adj. *p*‐value < .05. **(E)** Heat map of significant genes after transduction with crRaf1_crPkm2, crRaf1 and crPkm2 in Hepa1‐6 cells. **(F)** Heat map of downregulated energy metabolism genes after transduction with crRaf1_crPkm2 in Hepa1‐6 cells. **(G)** Systematic characterisation of the changes in the metabolic pathways caused by the pivotal downregulated genes after Raf1‐Pkm2 double knockout. **(H)** Kaplan–Meier curves depicting the association between cytotoxic T lymphocyte (CTL) levels and the overall survival of patients with HCC and high or low expression of Pkm2. Statistics are shown in the plot. **(I)** Kaplan–Meier curves depicting the effect of the Raf1_Pkm2 combination on the overall survival of patients with LIHC from TCGA databases. **(J)** Kaplan–Meier curves depicting the effect of the Raf1_Pkm2 combination on the overall survival of patients with LIHC from GEO databases. num(T): number of tumour samples.

To verify whether our identified combinations exhibited favourable translational potential for clinical efficacy, the performance of all 180 double‐knockout combinations was analysed based on the overall survival (OS) of patient samples from the TCGA database. Multiple gene pairs was identified to be significantly associated with overall survival (OS) in liver hepatocellular carcinoma (LIHC) patients, and low expression of Raf1‐Pkm2 was observed with increased OS in LIHC patients, suggesting that Raf1‐Pkm2 could be an excellent translational candidate for clinical efficacy (Figure 6A). To explore the molecular mechanisms involved in the regulation of HCC growth by the combinations we identified, we selected Raf1_Pkm2 gene pairs and performed RNA‐seq to analyse global transcriptional variation in single‐knockout and double‐knockout cells for Raf1_Pkm2. We observed that double knockout of Raf1_Pkm2 regulated fatty acid catabolic processes and accelerated energy metabolism, and that double knockout of Raf1‐Pkm2 significantly promoted apoptosis. Meanwhile, the results of double knockout were consistent with the pathways involved in the low expression of Raf1‐Pkm2 in the TCGA database (Figure 6B and C and S8A). Previous research has shown that glycolysis and fatty acid synthesis are abnormally active in HCC cells and tissues and are inextricably involved in tumour growth. Here, we analysed the gene cluster after Raf1‐ Pkm2 double knockout and revealed significant downregulation of key genes involved in glycolysis and fatty acid synthesis, leading to the stagnation of energy metabolism. This suggests that cell death in HCC induced by Raf1‐Pkm2 double knockout may be achieved through inhibition of the glycolysis and fatty acid synthesis pathways (Figure 6E–G). Overall, our results show that Raf1‐Pkm2 has reasonably good therapeutic potential and that the pathways involved in gene knockout are broadly similar to those involved in sorafenib treatment, reflecting congruence in genetics and pharmacology.

Furthermore, these two genes may be general targets for multiple tumours, especially Pkm2, which is highly expressed in most tumours (Figure 6D and S9). Low expression of Pkm2 may improve OS rate under high CTL (cytotoxic T lymphocytes) compared to high expression of Pkm2, showing good therapeutic potential for Pkm2 (Figure 6H). Kaplan–Meier curves from TCGA databases showed that patients with nonviral‐related HCC with low Raf1‐Pkm2 expression exhibited increased overall survival (Figure 6I). The Gene Expression Omnibus (GEO) database showed a similar therapeutic effect: low expression of Raf1‐Pkm2 significantly improved patient survival (Figure 6J). These findings suggest that the crosstalk between Raf1 and Pkm2 might be important in HCC development and that Raf1‐Pkm2 could be a good target for combination with FDA‐proved drugs. To verify whether Raf1‐Pkm2 could be clinically translated in the treatment of other cancers, we determined the effect of Raf1 and Pkm2 gene pairs on the survival rate of other tumours in the TCGA database and observed that the Raf1 and Pkm2 gene pairs were related to two other cancers, thymoma (THYM) and lung adenocarcinoma (LUAD), showing favourable clinical efficacy compared to single‐gene knockout (Figure S8B and C).

### 2.7 Tissue microarray confirmed clinical correlation between Raf1 and Pkm2

**FIGURE 7:**
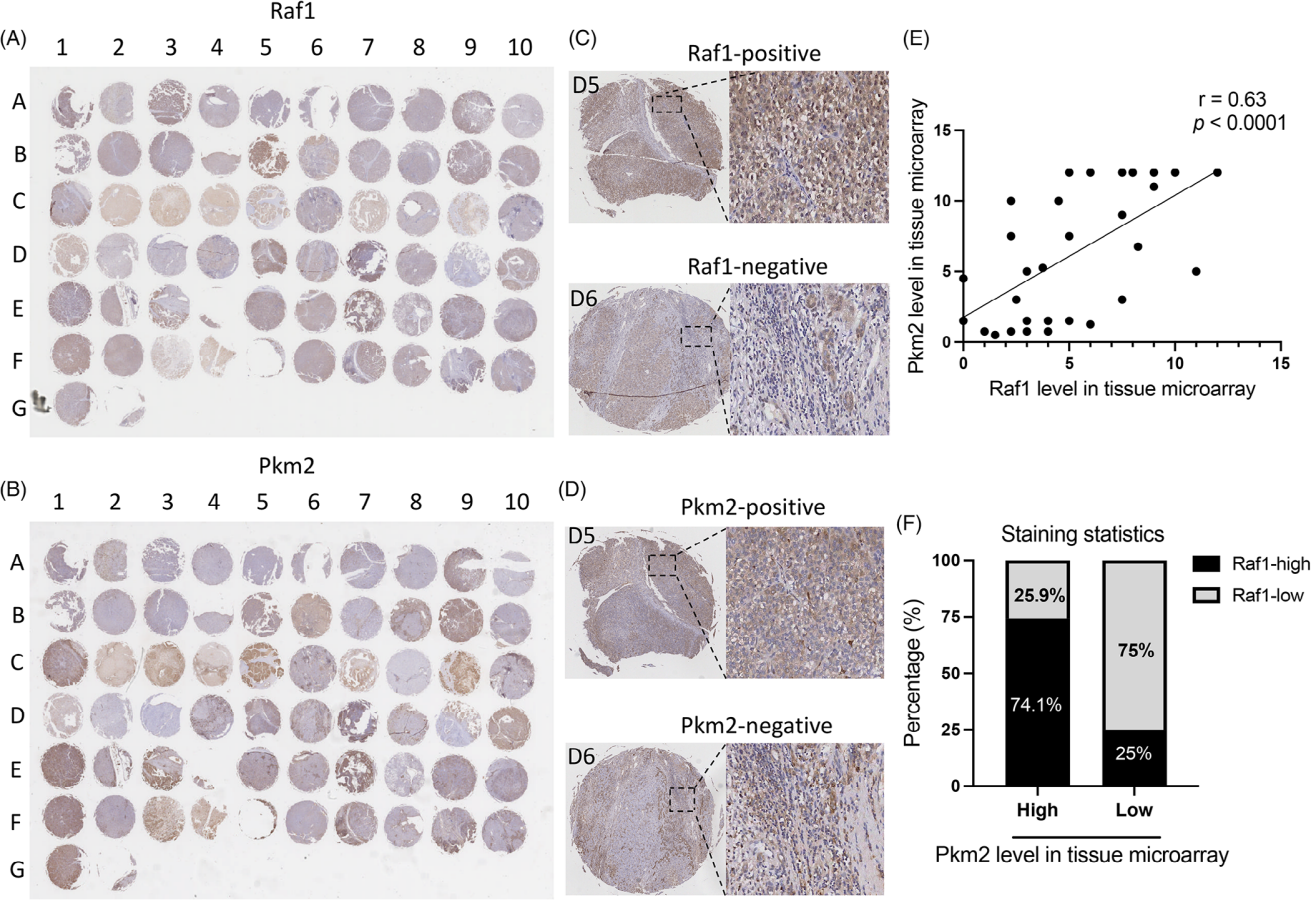
**Tissue microarray confirmed clinical correlation between Raf1 and Pkm2**. (A) Overall staining of Raf1 in the HCC tissue microarray and the tissue microarray contained 62 tissue samples for analysis. (B) Overall staining of Pkm2 in the HCC tissue microarray and the tissue microarray contained 62 tissue samples for analysis. (C) Representative staining of positive and negative immunostaining for the Raf1 in human HCC tissues. (D) Representative staining of positive and negative immunostaining for the Pkm2 in human HCC tissues. (E) Scatter plot compares the correlation between the expression levels of Raf1 and Pkm2 in tissue microarrays. Spearman correlation coefficient = 0.6317, *p* < .0001. (F) The bar graphs show the results of tissue microarray staining. There is a significant positive correlation between Raf1 and Pkm2 in tumour tissue.

To explore the clinical significance of the Raf1‐Pkm2 gene combination, we analysed the expression levels of Raf1, Pkm2 in patient samples and their correlation with clinicopathological features by tissue microarray. We calculated the staining scores (expression levels) of Raf1 and Pkm2 from the staining intensity and positivity of tissue microarray samples. We analysed the expression levels of Raf1 and Pkm2 in HCC tissues from 31 patients and found that the expression of both Raf1 and Pkm2 genes was upregulated in most of the HCC tissues, which was further determined by the paired Wilcoxon signed‐rank test. The expression levels of Raf1‐Pkm2 in tumour and adjacent tissues were statistically significant (Figure 7A–D and Table 1). To investigate the correlation between the expression of Raf1 and Pkm2 in clinical samples, we compared the expression levels of Raf1 and Pkm2 in all tumour tissue samples by Spearman correlation analysis, and the results showed that the correlation coefficients (*r*) = 0.63, and the correlation was statistically significant (Figure 7E). Statistics of stained samples showed that in samples with high Pkm2 expression, 74.1% of samples had high Raf1 staining intensity and positivity, whereas in samples with low Pkm2 expression, 75% of samples had low Raf1 staining intensity and positivity (Figure 7F). These data suggest a positive correlation between Raf1 and Pkm2 expression levels in tumour tissues. We then analysed the correlation between Raf1 and Pkm2 and clinicopathological characteristics by the rank‐sum test of two independent samples, and the results of this showed that both Raf1 and Pkm2 had a high correlation with pathological grades, suggesting that Raf1 and Pkm2 are cooperatively involved in the formation and development of HCC, and may become effective targets in the progression stage of HCC.

**TABLE 1:** Differential expression of Raf1 and Pkm2 in tumour tissues and adjacent tissues.

*n*	M (P_25,_ P_75_)		*Z* value	*p* Value
	Tumour tissues	Adjacent tissues		
Raf1 62	5.00 (2.50, 8.00)	1.50 (0.50, 3.00)	–3.446	<.001^*^
Pkm2 62	5.25 (1.50, 12.0)	0.5 (0.00, 1.50)	–4.229	.000^*^

* Statistically significant (*p *< .05).

M: median, P25: lower quartiles, P75: upper quartiles.

## 3 DISCUSSION

Off‐target effects are critical in CRISPR negative selection screens.^25^, ^26^ In CRISPR screening, off‐target effects can significantly affect screening accuracy and edit nontarget targets, affecting the screening results. In clinical applications, nonspecific cleavage caused by off‐target effects interferes with the normal regulatory function of nontargets, leading to serious side effects. Therefore, the off‐target rate is key in determining whether in vivo gene editing can achieve precision therapy. In our previous study, we identified a novel Cas12a orthologue with high gene‐editing efficiency, CeCas12a, which has high restriction of PAM sequences in vitro and in vivo and low off‐targeting in the PAM and other regions compared to AsCas12a and LbCas12a23. In this study, using guide‐seq, deep sequencing, and T7E1 assays, CeCas12a was observed to be a powerful Cas12a nuclease with the lowest off‐target effects, high gene‐editing efficiency, and good reliability between different repeats in the SOCA double‐knock library. A SOCA library was constructed to perform double‐knockout screening in Hepa1‐6 cells, and several novel gene pairs influencing HCC carcinogenesis were identified. Genetic interactions were highlighted using two mathematical models that showed reasonably good performance and were further checked by in vitro and in vivo assays. The specific signalling pathways involved were further analysed.

Usually, for the design of traditional libraries, the number of sgRNAs that target the library genes is vital to achieving efficient screening. However, fewer crRNAs per gene provide more information, which is critical for in vivo screening.^27^ Moreover, it is difficult to cover large combinations of whole human genes for double‐ or multiple‐knockout screens. A rapid and specialised library would be a good choice in the future. Therefore, in our study, to ensure the accuracy of library screening for combinations of FDA‐approved drug targets, we aimed to test Cas12a nucleases with low off‐target rates and high gene‐editing efficiency for the designed SOCA library. Furthermore, compared with traditional double‐knockout screening using multiple arrays for targeting each pair,^28^, ^29^, ^30^ the SOCA library is greatly improved due to its convenience in design, and the on‐targeting and off‐targeting of library crRNAs can be rapidly checked before double‐knockout screening to avoid possible false signals.

Chow et al.^15^ performed CRISPR‐Cas12a crRNA array screening under positive selection for increased cell growth to assess the metastatic potential of mouse tumours and observed that the identified combinations contained stationary crRNAs. Under negative selection for cell growth inhibition in our screening system, stationary crRNA was observed. By comparing the results of CeCas12a‐ and Lb2Cas12a‐mediated library screening, we observed that CeCas12a identified significantly more gene combinations than Lb2Cas12a, which was closely related to the efficient gene editing efficiency and lower off‐target rate of CeCas12a. The SOCA library screening results showed that multiple combinations were associated with Raf1 knockout, and Raf1‐NTC emerged as a significant outlier in the linear regression analysis of the cell pool versus tumour. Although Raf1 is a stationary crRNA in our identified combinations, a single knockout of Raf1 had a minimal effect on the relevant signalling pathways using RNA‐seq, which aligns with the result of stationary crRNA research.^16^

In order to ensure the reliability of the screening results, we identified interactions in gene pairs using mathematical models established in previous studies. By analysing the combinational effect of gene pairs based on pairs versus corresponding single genes using the two‐sided Wilcoxon rank‐sum test and significant outliers in linear regression based on expected DKO versus observed DKO, we observed a highly significant interaction between Raf1 and Pkm2. In addition, we determined the variation in the abundance of DKO, SKO and NTC‐NTC of the Raf1‐Pkm2 gene pair in cell pools and tumour screens. The abundance of the Raf1‐Pkm2 gene pair was significantly reduced within the tumour, suggesting progressive selection pressure as the cells pool to form the primary tumour. In response to this pressure, the most pronounced variation in the abundance of Raf1‐Pkm2 was observed, confirming a genetic interaction with Raf1‐Pkm2. In the RNA‐seq experiment, the combinatorial knockout of Raf1_Pkm2 significantly regulated cellular kinase signalling pathways and gene expression compared to Raf1_NTC and Pkm2_NTC. Pathway enrichment analysis correlating with Raf1 or Pkm2 expression suggested that low expression of Pkm2 upregulates multiple small‐molecule catabolic processes and low expression of Raf1 downregulates energy transport activities. Pathway enrichment analysis of Hepa1‐6 cells and the TCGA database suggested that double knockout or low expression of Raf1‐Pkm2 significantly disturbed fatty acid biosynthesis and ATPase‐dependent transmembrane transporter activity, both of which have been reported in previous studies to be closely associated with HCC development.^31^, ^32^, ^33^ Therefore, FDA‐approved combinations of drugs that target both Raf1 and Pkm2, such as sorafenib and benserazide, may be promising for clinical liver cancer treatment, as confirmed by our combination treatment in multiple hepatocellular carcinoma cells. Although the FDA drug treatment experiments did not show a highly significant synergistic effect, the occurrence of such a situation is related to the sensitivity of the drugs to different hepatocellular carcinoma cell lines. Our results indicated that the combination of the two drugs was effective in MHCC97L cells, which may be because the metastatic potential of MHCC97L cells is higher than that of HepG2, Hep3B and Huh7 cells, and the intrinsic differences in protein expression make MHCC97L cells more sensitive to drugs. In addition, there was no direct equivalence between gene knockout and FDA drug treatment during the phenotype validation, and the latter exerted a more complex effect. Benserazide, an FDA‐approved Pkm2 inhibitor that inhibits peripheral dopa decarboxylase for the treatment of Parkinson's disease, has a weak effect on the induction of apoptosis; however, our results show that benserazide is still involved in combination therapy with Raf1. We have established double‐knockout and single‐knockout nu/nu mice tumour models of Raf1‐Pkm2 and showed a significant reduction in tumour size with the double knockout of Raf1 and Pkm2, whereas single knockout did not result in a significant reduction in tumour size. Genetic knockout of Raf1‐Pkm2 fits better with the validation of our SOCA library screening results for the HCC model. In addition, the synergistic effect of the Raf1‐Pkm2 combination was confirmed using specific inhibitors, indicating good translational potential for clinical therapy. In clinical samples, tumour microarray experiments confirmed a positive correlation between the expression levels of Raf1 and Pkm2 in tumour tissue, suggesting that Raf1‐Pkm2 may serve as a synergistic therapeutic target for HCC development.

Moreover, compared with the traditional double‐knockout library, our rapid lab‐made library construction method with high‐fidelity CeCas12a could easily be expanded to achieve triple‐ or multiple‐knockout screening of thousands or tens of thousands of gene combinations with low off‐target disturbances. One reality that may limit the widespread use of SOCA libraries is that each combination consists of a single crRNA array, which requires significant cleavage of each crRNA in the array to achieve accurate and efficient library screening. Therefore, the cleavage efficiency of each crRNA target needs to be determined using deep sequencing or the T7E1 assay before library screening, which is a substantial effort when synthesising large libraries containing hundreds of targets, and predictive crRNAs with high cleavage activity may be a good choice. In the future, double‐knockout screening in patient‐derived primary tumour cells will be an important way to link SOCA libraries to clinical translation, as primary tumour cells allow for the preservation of most genetic and epigenetic variants in HCC, thus ensuring the screening of reliable clinical drug targets. Unlike the traditional random combination screening within a gene set, through our SOCA library, different gene sets involved in different pathways or biological processes can be easily constructed to focus on cross‐talk and explore the interaction between different pathways or biological processes, which is necessary to explore the complex regulatory pathways in HCC.

## 4 MATERIALS AND METHODS

### 4.1 Cell culture

Hepa1‐6, a murine HCC cell line, was derived from CCTCC (China Center for Type Culture Collection).

Hepa1‐6 was transduced with Lenti‐EFS‐CeCas12a‐Puro or Lenti‐EFS‐Lb2Cas12a‐Puro to generate CeCas12a‐ or Lb2Cas12a‐positive cells, respectively.

All cell lines were incubated at 37°C with 5% CO_2_ in DMEM medium supplemented with 15% FBS and 1% penicillin‐streptomycin.

### 4.2 Tissue microarray analysis

The tissue microarrays containing 31 pairs of tumour tissues and paired‐adjacent tissues were purchased from Shanghai Outdo Biotech. To calculate an overall score (expression level), we multiplied the staining intensity of the tissue microarray samples by the positivity rate. Statistical differences in the expression of Raf1 and Pkm2 in tumour and adjacent tissues were analysed by paired Wilcoxon signed rank test, and the results are shown in Table 1. The correlation between the expression level of Raf1 and Pkm2 in tumour tissues and clinicopathological characteristics was analysed by two independent samples rank‐sum test, and the corresponding results are shown in Table 2.

**TABLE 2:** Correlation between Raf1 and Pkm2 expression and clinicopathological characteristics in hepatocellular carcinoma.

			Raf1		Pkm2	
	Variables	*n*	*Z* value	*p* Value	*Z* value	*p* Value
Age (year)	≤ 60 >60	24 7	–1.302	.193	–0.167	.867
Sex	Male Female	26 5	–0.108	.914	–0.815	.415
Pathological grade	I II/ III	11 20	–2.482	.013^*^	–2.734	.006^*^
Tumour size	≤ 5 cm > 5 cm	9 14	–0.758	.449	–0.572	.567

* Statistically significant (*p *< .05).

### 4.3 Design of the SOCA library

First, the top 15 kinase genes and the top 12 metabolism‐associated genes were selected based on three aspects. (1) We collected all significant oncogenes implicated in the HCC carcinogenesis and progression from GDAC (http://gdac.broadinstitute.org/). (2) The collected genes were compiled and sorted into two categories: kinase genes and metabolism‐associated genes. (3) Identification of molecular targets for FDA‐approved drugs by using the Drugbank database (https://www.drugbank.ca/drugs). We acquire all possible CeCas12a and Lb2Cas12a spacers by analysing shared exon sequences of all transcripts for 27 candidate genes from CRISPOR (RRID:SCR_015935) (all spacers have 5′‐TTTV PAMs and 20 bp guides). The standard for selecting optimal spacers is to filter out all alignments containing mismatches in the last 3 base pairs (corresponding to the PAM of Cas12a) and to disregard any mismatches in the fourth to last base pair. Finally, we verified the optimal spacers of 27 candidate genes using the T7E1 assay.

We designed a pair of overlapping complementary primers on two crRNA spacers and spliced the two crRNA spacers with complementary ends by SOE PCR to amplify long DNA fragments, the latter containing core fragments composed of spacer and DR. We then confirmed the size of the product by agarose gel electrophoresis and performed gel extraction to extract the DNA fragment and perform infusion cloning with the PLL3.7 blast vector (Figure S1B). The recombinant product was transformed into competent cells. After Maxi plasmid extraction, deep sequencing was performed to verify the coverage of the library.

### 4.4 Amplification and sequencing of the SOCA library

We conducted a two‐step PCR approach to perform the SOCA library readout. First, in the 1st PCR, we used an appropriate amount of genomic DNA as a template to ensure the coverage of the SOCA library. 15 µg of gDNA per sample was divided into 10 separate PCR reactions. In the first PCR, the sgRNA‐containing region was amplified using primers specific for the PLL3.7 (RRID:Addgene_11795) vector using Phanta MAX Super‐Fidelity DNA Polymerase P505 (Vazyme) with thermocycling parameters: 95°C for 3 min; 15 cycles of 95°C for 15 s, 60°C for 10 s, 72°C for 10 s; and 72°C for 5 min.

mU6 recovery Fwd ACAGCACAAAAGGAAACTCACCCTAACTG

mU6 recovery Rev CGATACCGTCGACCTCGAGGGTCACCGCG

In the 2nd PCR, we mixed the products of the 1st round PCR from each biological replicate, then used 1 µL of well‐mixed 1st PCR products as the template for amplification using index primers with thermocycling conditions as 95°C for 30 s; 15 cycles of 95°C for 15 s, 60°C for 10 s, 72°C for 10 s; and 72°C for 5 min.

Index 5 Fwd

AATGATACGGCGACCACCGAGATCTACACNNNNNNNNACACTCTTTCCCTACACGACGCTCTTCCGATCTTATCCCTTGGAGAAAAGCCTTGTTTG (index NNNNNNNN)

Index 7 Fwd

CAAGCAGAAGACGGCATACGAGATNNNNNNNNGTGACTGGAGTTCAGACGTGTGCTCTTCCGATCTCCTCGAGGGTCACCGCGCGTTAAC (index NNNNNNNN)

The 2nd PCR products were purified using 2% agarose gel. The purified SOCA library was sequenced with HiSeq 4000 systems (Illumina) with 150 bp paired‐end read length.

### 4.5 Double‐knockout screening data processing and analysis

Use read1 and read2 for statistics

1. First, by determining the overlap sequence of read1 and read2, merge read1 and read2 (merged_read) on the condition that overlap length must be more than 30 bp.
2. Search for library sequences in the merged_read, and count the times each sequence of the library which searched in merged_read.

Raw crRNA array counts were converted to reads per million (rpm) to normalise the number of reads in each sample. For cell pools, we used FC (fold‐change) value to represent the ratio of the final crRNA rpm to the initial crRNA rpm, and FC value was further converted into LFC (log_2_ FC) value to analyse the variation of crRNA abundance. For the primary tumour, to identify the gene interactions, linear regression was performed on tumour versus cell pools and the outlier test function from the ‘car’ R package was used to calculate the studentised residuals and *p*‐values of the linear regression. Finally, *p*‐values for multiple comparisons were adjusted using Benjamini–Hochberg.

### 4.6 Identification of gene pair interactions

Based on the abundance distribution of the SOCA library in cells and tumours, we applied two mathematical models more established in previous studies to characterise the potential interactions of gene pairs in our library.^15^, ^34^ First, the read counts of samples was converted to RPM and the synergistic effect of gene pairs based on pairs versus corresponding single genes was analysed using the two‐sided Wilcoxon rank‐sum test as a way to identify DKO gene pairs with statistical significance compared to SKO gene pairs. Second, expected DKO versus observed DKO under NTC‐NTC normalisation was analysed by linear regression model to identify enrichment based gene interactions by identifying significant outliers.

Observed DKO enrichment = (*D*_t_/*D*_c_) × (*N*_c_/*N*_t_).

Expected DKO enrichment = Gene A + Gene B enrichment = (*A*_t_/*A*_c_) × (*N*_c_/*N*_t_) + (*B*_t_/*B*_c_) × (*N*_c_/*N*_t_).

*D*_t_ = median abundance for DKO gene pairs observed in tumour screen.

*D*_c_ = median abundance for DKO gene pairs observed in cell screen.

*A*_t _= median abundance for SKO gene A observed in tumour screen.

*A*_c_ = median abundance for SKO gene A observed in cell screen.

*B*_t _= median abundance for SKO gene B observed in tumour screen.

*B*_c_ = median abundance for SKO gene B observed in cell screen.

*N*_t_ = median abundance for NTC‐NTC observed in tumour screen.

*N*_c_ = median abundance for NTC‐NTC observed in cell screen.

### 4.7 Targeted deep‐sequencing

After 72 h of transduction, cells were collected and lysed by using Animal Tissue Lysis Component. On‐target sites were amplified using Phanta MAX Super‐Fidelity DNA Polymerase P505 (Vazyme) with thermocycling parameters: 95°C for 3 min; 38 cycles of 95°C for 15 s, 60°C for 10 s, 72°C for 30 s; and 72°C for 5 min. Each on target fragment is approximately 150 bp. Primers used for deep sequencing were listed in Data file S1. PCR products were purified with AFTMag NGS DNA Clean Beads (ABclonal RK20257). The purified pooled library was sequenced on HiSeq 4000 systems (Illumina) at 150 bp paired‐end read length.

### 4.8 GUIDE‐seq

The fundamental mechanisms of GUIDE‐seq is that dsDNA tag is integrated into the fracture site of the cell genome, amplified with specific tag primers and obtain sequence near on target and off target by sequencing.^35^ To explore the off‐target effect of CeCas12a, AsCas12a and Lb2Cas12a in Hepa1‐6 cells, we performed GUIDE‐seq to detect off‐target sites for all candidate genes in the SOCA library. We took 1 µg of each of 27 crRNA plasmids (concentration greater than 1 µg/µL) for equal mass mixing, and digested Hepa1‐6 cells with a growth density of about 80% into 2 × 10^7^ cells /mL in a 10 cm dish on the day of electroporation. Electroporation is performed according to the Neon™ transfection system (invitrogen™) operation manual. The number of cells in a 10 µL of electroporation system was 2 × 10^5^. Each Cas12a (RRID:Addgene_188492) editing protein was electrocuted in parallel for 5 repetitions, and 1000 ng of Cas12a plasmid and 500 ng of crRNA mixed plasmid were added to each well. After electroporation, the cells were dropwise added to complete medium without antibiotics and cultured at 37°C under 5% CO_2_. 72 h after incubation, FastPure® Cell/Tissue DNA Isolation Kit (Vazyme) was used to extract and purify genomic DNA. Subsequently, we used Hieff NGS® Fast‐Pace DNA Fragmentation Reagent to break up 1000 ng of genomic DNA, conducted end‐repair, and added A‐tailing. Finally, we used T4 DNA ligase to ligate the universal adaptor and amplification with Tag‐specific primers to obtain library. After purification and quantification for the GUIDE‐seq library, the library was sequenced on an Illumina instrument. GUIDE‐seq data were analysed using the GUIDE‐seq analysis software (http://www.jounglab.org/guideseq).

### 4.9 T7E1 assays

At 72 h post‐transfection, cells were collected for genomic DNA extraction using the Animal Tissue Direct PCR Kit. PCR amplified the genomic region flanking the Cas12a target site of each gene, and products were purified using PCR Product Purification Kits. A total of ∼300 ng of purified PCR amplicons were denatured, reannealed and digested with T7E1 (Vazyme). The reaction mixtures were run on 2% agarose gels after 20 min incubation at 37°C. Finally, the gels were imaged using ChemiDocTM XRS+ and analysed according to band intensities.

### 4.10 Lentiviral library transduction

Three plasmid systems were used to transfer psPAX2, PMD2.G and SOCA library plasmid DNA into HEK293T cells at a ratio of 1:1:2 in the presence of Hieff Trans Liposomal Transfection Reagent. Eight hours after transfection, the transfection medium was replaced with complete DMEM medium containing 15% FBS. At 48 and 72 h post‐transfection, the virus supernatant was collected and centrifuged at 1500 × *g* for 15 min to coarsely remove cell debris. The virus supernatant was then filtered through a 0.45 µm filter membrane, and the filtered virus supernatant was concentrated with lentivirus concentrated solution (CAT: Hg‐lpkc050, HonorGene, Changsha) in a volume ratio of 4:1 for virus supernatant: concentrated solution and placed in an ice bath overnight. The mixed solution was centrifuged at 1500 × *g* for 45 min to collect the virus precipitate, and an appropriate volume of serum‐free medium was added to dissolve the virus precipitate. After viral titres were determined by relative quantitative PCR, target cells were infected at a lower multiplicity of infection (MOI = 0.2) with 8 µg/mL Polybrene (CAT: 40804ES76, Yeasen, Shanghai). The viral medium was replaced with complete DMEM medium 24 h after infection. Add 2 µg/mL Puromycin and 10 µg/mL Blasticidin to perform target cell selection at 48 h post‐infection.

### 4.11 SOCA library screening in a mouse HCC model

SOCA library lentiviruses transduction requires appropriate MOI and coverage. Here, we used SOCA library lentiviruses to infect 1 × 10^7^ cells with a lower MOI to reach 1000 × library coverage. Lentiviral SOCA library‐transduced target cells were cultured under a selection pressure of 10 µg/mL blasticidin for 7−14 days. SOCA library‐transduced cells were injected subcutaneously into the right fossa axillaris of *nu/nu* mice at 4 × 10^6^ cells per mouse. Based on tumour growth, we selected *nu* */nu* mice on day 10 and day 18 to extract the tumour genome to conduct library construction for deep sequencing, and the variation of crRNA abundance between the two time periods was analysed. Animal experiment selected *nu / nu* female mice, aged 5 weeks and weighing 18–20 g per mouse. The mice were randomly divided into four groups with six mice in each group. Mice that tumour volume exceed 2000 m^3^ were excluded. Moreover, tumours over 2 cm in length were excluded for this study.

### 4.12 Genomic DNA extraction

#### Digestion and lysis of tumour tissue

100 mg of mechanically broken tissue was placed in 15 mL centrifuge tube, 1.1 mL of Buffer GA and 20 µL of Proteinase K were added, mixed by vortex, and the tissue was incubated at 55°C bath overnight to achieve thorough enzymolysis. Then 10 µL of RNase solution was added to the digestion solution, mixed upside down, and incubated at room temperature for 60 min to eliminate residual RNA. Then centrifuged at 4000 × *g* for 3 min, transferred the supernatant to a new 1.5 mL centrifuge tube, added 1.2 mL Buffer GB to the digestive solution, vortexed and mixed at the highest speed for 20 s, and then bathed at 70°C for 10 min. At the end of the water bath, add 900 µL of absolute ethanol to the digestive solution and vortexed for 20 s. The mixture was transferred to the gDNA column several times and centrifuged at 12 000 rpm for 1 min. Washing Buffer A and Washing Buffer B were added and centrifuged for 1 min, followed by empty tube centrifugation for 2 min. Place the gDNA column in new 1.5 mL centrifuge tube, add 150 µL of Elution Buffer preheated to 70°C in the centre of the membrane of the gDNA column, placed it at room temperature for 3 min, then centrifuged it at 12 000 rpm for 1 min, stored the DNA at −20°C.

#### 4.12.1 Digestion and lysis of cultured cells

About 5 × 10^6^ cells were centrifuged at 400 × *g* for 5 min to collect the cells, then the supernatant was discarded. 220 µL PBS, 10 µL RNase Solution and 20 µL Proteinase K were added to the cell precipitation, and the cells were resuspended and left at room temperature for 15 min. Then, 250 µL Buffer GB was added to the cell resuspending, and the mixture was vortexed for 20 s, followed by a 65°C water bath for 30 min. Add 180 µL of absolute ethanol to the digestion solution and vortex the SEC. The following steps are the same as the digestion and lysis of tumour tissue.

### 4.13 TCGA analyses

The normalised expression matrix and paired survival data was selected from the TCGA‐LIHC transcriptome data downloaded from the web page. Clinical data for paired samples were downloaded from previous study.^36^ HBV or HCV samples that were detected in both clinical and transcriptome data were removed from the TCGA‐LIHC samples, and then selected 27 candidate genes from SOCA library. The Mann–Whitney *U* test of IPython (RRID:SCR_001658, version 1.9.1) package scipy was used to calculate the differential expression of Raf1, Top2A and Pkm2 between tumour samples and normal liver samples. Then we calculated the mean of normalised expression fpkm_uq_unstranded for the two genes in each gene pair derived from 180 gene pairs in the experiment, and the samples were divided into two groups of cutoff‐high (75–100) and cutoff‐low (0–25) by the mean value. The KM method in the R package survival (version 3.5‐5) was used to calculate the survival differences between two groups and denoted as the effect of the gene pair on survival rates. Finally, the effects of single gene on survival were calculated using a similar approach.

For each gene pair, the *p* value of the effect of that gene pair on survival was used to be divided by the *p* value of the effect of their single gene on survival, and the resulting value was then added 1 and shown it in logarithm (np.log1p), and then presented as scatter plots after removing outlier value. Gene pairs with both values greater than 1 and unfavourable for survival rate are marked in red, and gene pairs with both values greater than 1 and favourable for survival are marked in blue. Others are marked in grey.

Based on the expression level of Pmk2, the HBV and HCV negative samples in TCGA‐LIHC were divided into PKM‐high and PKM‐low groups, and the cytotoxicity score obtained by MCPCOUNTER was calculated for assessing the influence on survival rate, respectively.

We calculated the correlation of Raf1 and Pkm2 expression with other genes in HBV and HCV negative samples in TCGA‐LIHC. For the gene clusters significantly related to a single gene, the pathway enrichment calculated by the R package clusterProfiler (RRID:SCR_016884, version 4.6.2) was recorded as the enrichment pathway of a single gene, and for the gene clusters significantly positively or negatively correlated with gene pair was recorded as the pathway affected by the two genes jointly.

### 4.14 RNA‐seq

#### RNA extraction and RNA Library construction

We efficiently extracted high‐purity total RNA from cells with knockout of the target gene using MolPure® Cell/Tissue Total RNA Kit. After quantification for total RNA using a microplate reader, 1 µg of total RNA was used for Library construction work using Hieff NGS® Ultima Dual‐mode RNA Library Prep Kit (CAT: 12252ES08, Yeasen) for Illumina® following the manufacturer's protocol.

#### Data processing for RNA‐seq

The original high‐throughput raw data were first subjected to trimmomatic (version 0.39) for quality control and adapter trimming, and the trimmed reads shorter than 50 bp were filtered. The clean data were then mapped to the mouse genome (GRCm39) using STAR (version 2.7.10b) with default parameters, and the transcriptome was generated based on the mouse Refseq annotation downloaded from the UCSC database. Read counts were calculated using subread (version 2.0.1), and gene expression was quantified as fragments per kilobase of exon model per million mapped fragments (FPKM) values, and genes with FPKM > 0.5 were considered to be expressed.

#### Identification of differentially expressed genes

RNA‐seq read counts were used to identify DEGs using the DESeq2 package in R version 4.2.1. Genes were considered as DEGs only if the absolute value of the log_2_‐transformed fold‐change was greater than 1 with both adjusted *p* values less than 0.05. GO analysis and KEGG analysis were performed using enrichGO and enriKEGG, respectively, with default parameters.

### 4.15 Colony formation assay

For colony formation assays, we seeded HCC cells in 6‐well plates (1–2 × 10^4^ cells per well), and treated with GW5074 (Raf1 inhibitor) and Shikonin (Pkm2 inhibitor) for 24 h. After that, the medium was then replaced with fresh medium every 2 days to maintain growth. Incubation continued for 2 weeks. Cells were fixed with methanol for 30 min, stained with 0.1% crystal violet solution for 10 min and photographed.

### 4.16 Statistics analysis

Statistical analysis was performed using GraphPad Prism (RRID:SCR_002798) software version 9.0. *p*‐Values for Pearson's correlation analysis between two groups were determined by the two‐tailed test. *p*‐Values for linear regression analyses were determined by two‐sided outlier test. *p*‐Values for comparisons between multiple groups were determined by two‐way ANOVA. *p* < .05 was used to denote statistical significance and error bars was used to denote the standard error of the mean.

## AUTHOR CONTRIBUTIONS

L.Y. conceived and supervised this work. Q.C. performed the SOCA library plasmid construction. Q.C., M.‐H.P., P.C. performed negative selection screening in cell pools and data analyses. Z.‐H.Z., J.L. performed animal experiments. Y.‐K.W., S.‐Q.L., B.‐W.J. performed bioinformatics analyses. Z.‐Q.S., B.‐X.H. performed the RNA‐seq library construction for Raf1_Pkm2 knockout in Hepa1‐6 cells. Q.C., L.Y., C.P. wrote and reviewed the manuscript.

## CONFLICT OF INTEREST STATEMENT

The authors declare that they have no competing interests.

## FUNDING INFORMATION

This work was supported by the National Key R&D Program of China (2022YFA1303500), the National Natural Science Foundation of China (32171210, 32371271 and 32101196), the China Postdoctoral Science Foundation (2021TQ0253, 2022M712468) and the Fundamental Research Funds for the Central Universities (2042022kf1189).

## Supporting information


**Figure S1: The confirmation of activities for predicting crRNAs and SOCA library products. (A)** The activities for predicting crRNAs of 27 library candidate genes were assessed using the T7E1 assay. **(B)** The SOE PCR product was confirmed using agarose gel electrophoresis.

**Figure S2: Activities of CeCas12a, AsCas12a and Lb2Cas12a on four candidate targets. (A)** Full images of Figure 1A. Two independent transduction replicates were done, and activities were assessed using theT7E1 assay. **(B)** Full images of Figure 1B. Boxes are shown in Figure 1A and C.

**Figure S3: Indels were induced by CeCas12a in Hepa1‐6 cells. (A)** The number of reads in input fastqs after preprocessing and alignment with amplicons. **(B)** Frequency distribution of alleles with indels (blue) and without indels (red). **(C)** Frequency of insertions (red), deletions (purple) and substitutions (green) across the entire amplicon, including modifications outside of the quantification window.

**Figure S4: Genome‐wide specificities of Cas12a orthologue‐matched crRNAs targeting three endogenous sites. (A)** On‐targets and off‐target sites for AsCas12a, CeCas12a and Lb2Cas12a with crRNAs targeting three endogenous sites determined using GUIDE‐seq in 293T cells. **(B)** Summary of the detailed number of off‐target sites identified using GUIDE‐seq for AsCas12a, CeCas12a and Lb2Cas12a with crRNAs targeting three endogenous sites.

**Figure S5: Parallel library‐mediated double‐knockout screening confirmed the rationality of SOCA library construction. (A)** Schematic describing the design for the parallel library. **(B)** Schematic of parallel library‐ mediated combinatorial screening in vitro. **(C)** A density plot depicting the abundance distribution of a parallel library. NGS sequencing determined 100% library coverage, consisting of 180 crRNA arrays. **(D)** Scatter plot depicting the abundance of the SOCA library and parallel library in the plasmid (*n* = 1) and averaged cell pools (*n* = 3). **(E)** The scatter plot compares the screening results for the parallel and SOCA libraries in CeCas12a‐positive cells. **(F)** The scatter plot compares the parallel and SOCA library screening results in Lb2Cas12a‐positive cells.

**Figure S6: Abundance analysis of the SOCA library in plasmid, cells and primary tumours. (A)** Heatmap that shows the Spearman's correlation coefficients for crRNA array log2 rpm abundance between SOCA plasmid library (*n* = 1), SOCA library transduced cells (*n* = 3 cell replicates) and primary tumours (*n* = 5‒6 nu/nu mice). **(B)** Tukey's boxplots showing the distribution of crRNA array log2 rpm abundance between SOCA plasmid library (*n* = 1), SOCA library transduced cells (*n* = 3 cell replicates), and primary tumours (*n* = 5‒6 nu/nu mice).

**Figure S7: Effect of specific compounds of Raf1 and Pkm2 on the activity of HCC cells. (A)** Quantitative analysis of GW5074 in combination with shikonin after treatment of hepatocellular carcinoma cells in vitro. **(B)** Shikonin in combination with GW5074 inhibited the proliferation of Hep3B, MHCC97L, HepG2 and Huh7 cells. **(C)** Long‐term colony formation assays show that GW5074 combined with shikonin exhibits synergistic inhibition in Hep3B and Huh7 cells. (D) Quantitative analysis of benserazide in combination with sorafenib after treatment of hepatocellular carcinoma cells in vitro. (E) Quantitative analysis of ZM 336372 in combination with shikonin after treatment of hepatocellular carcinoma cells in vitro.

**Figure S8: The GO enrichment analysis for the single knockout of Raf1 and Pkm2 in the TCGA database and Kaplan–Meier curves depicting the effect of the Raf1_Pkm2 combination on overall survival for other cancers. (A)** Pathway enrichment of genes negatively correlated with Raf1 expression and Pkm2 expression; representative KEGG pathways analysis showed that DEGs were enriched in downregulated gene sets. **(B)** Overall survival of patients with THYM under high or low expression for Raf1, Pkm and Raf1_Pkm2 from the TCGA database. **(C)** Overall survival of patients with LUAD under high or low expression for Raf1, Pkm and Raf1_Pkm2 from the TCGA database. THYM: thymoma; LUAD: lung adenocarcinoma.

**Figure S9: Boxplots of Raf1 and Pkm2 expression in a variety of tumour samples from the TCGA database. (A)** Boxplots of Raf1 expression in STAD, LUAD and BRCA samples. **(B)** Boxplots of Pkm2 expression in STAD, LUAD and BRCA samples. STAD: stomach adenocarcinoma; LUAD: lung adenocarcinoma; BRCA: breast cancer; num(T): number of tumour samples.

## Data Availability

All raw sequencing datasets are available in the GEO repository under the accession number GSE236831.
