# Supplementary figures and images for: High‐fidelity CRISPR/Cas12a dual‐crRNA screening reveals novel synergistic interactions in hepatocellular carcinoma
Source: Clin Transl Med. 2024 Jul 28;14(7):e1758. doi: 10.1002/ctm2.1758 (PMC11283585; doi:10.1002/ctm2.1758)

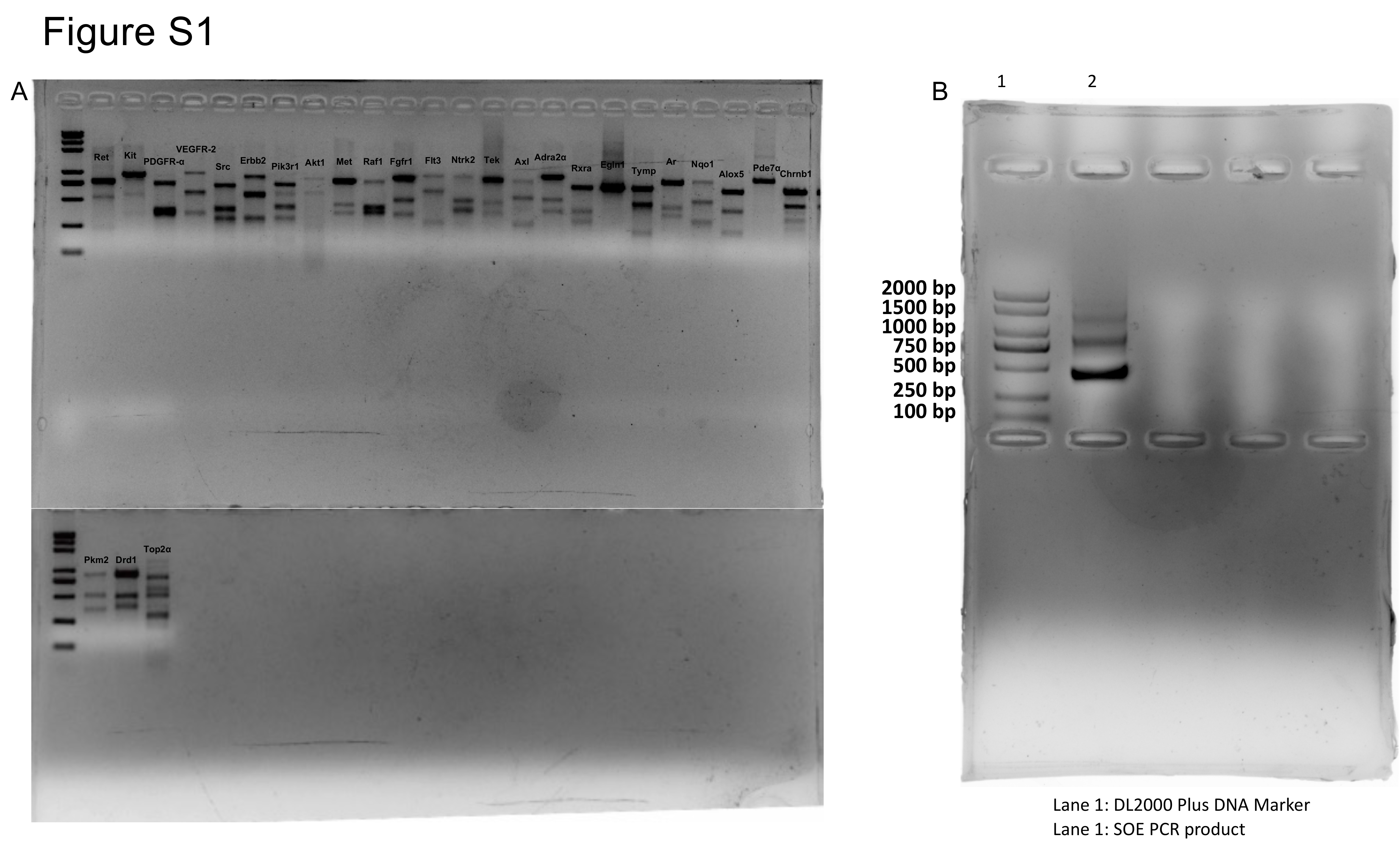

Supplement: Supplementary file 1 — Figure S1: The confirmation of activities for predicting crRNAs and SOCA library products. (A) The activities for predicting crRNAs of 27 library candidate genes were assessed using the T7E1 assay. (B) The SOE PCR product was confirmed using agarose gel electrophoresis. [file CTM2-14-e1758-s004.tif]

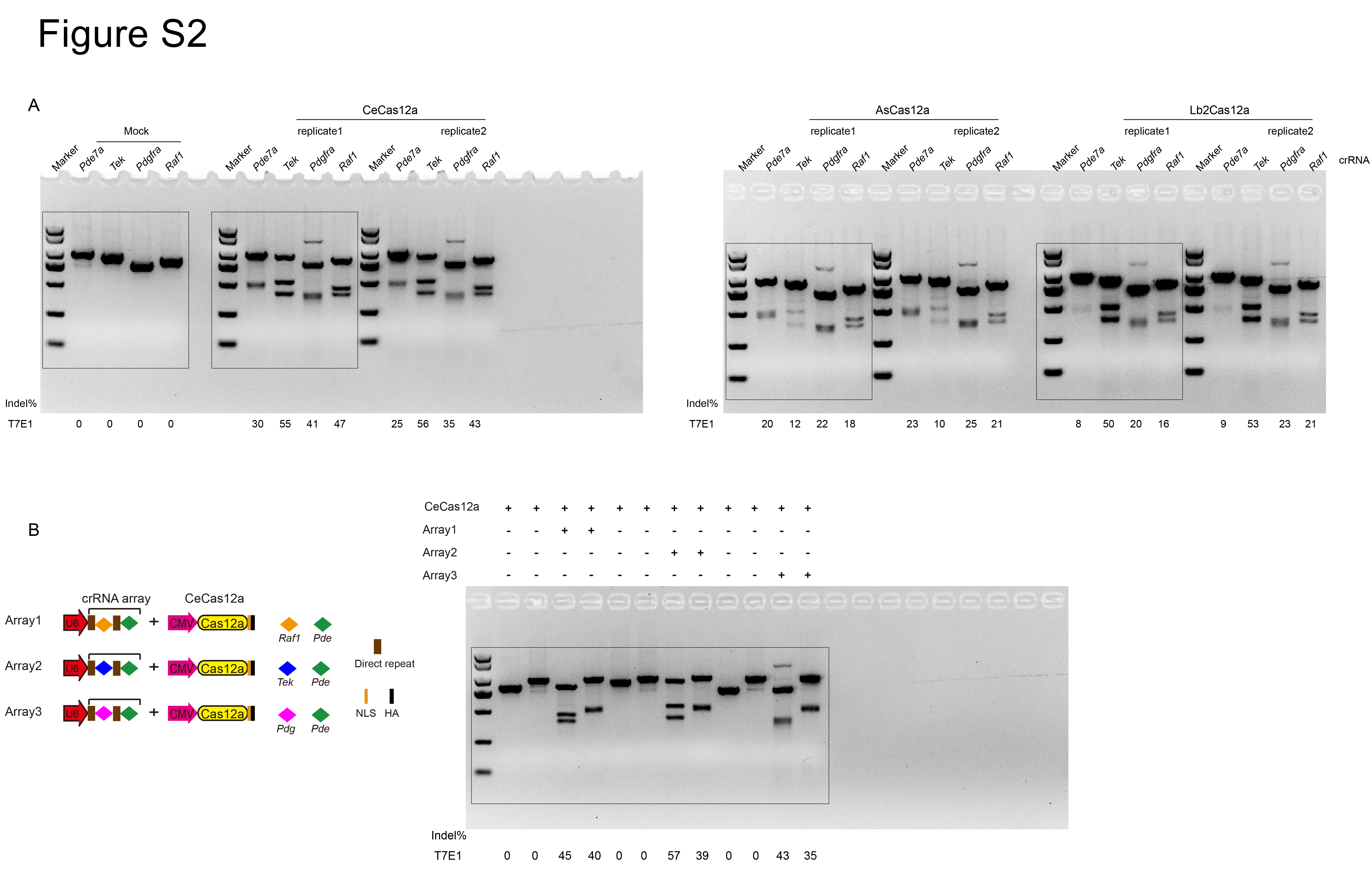

Supplement: Supplementary file 2 — Figure S2: Activities of CeCas12a, AsCas12a and Lb2Cas12a on four candidate targets. (A) Full images of Figure 1A. Two independent transduction replicates were done, and activities were assessed using theT7E1 assay. (B) Full images of Figure 1B. Boxes are shown in Figure 1A and C. [file CTM2-14-e1758-s007.tif]

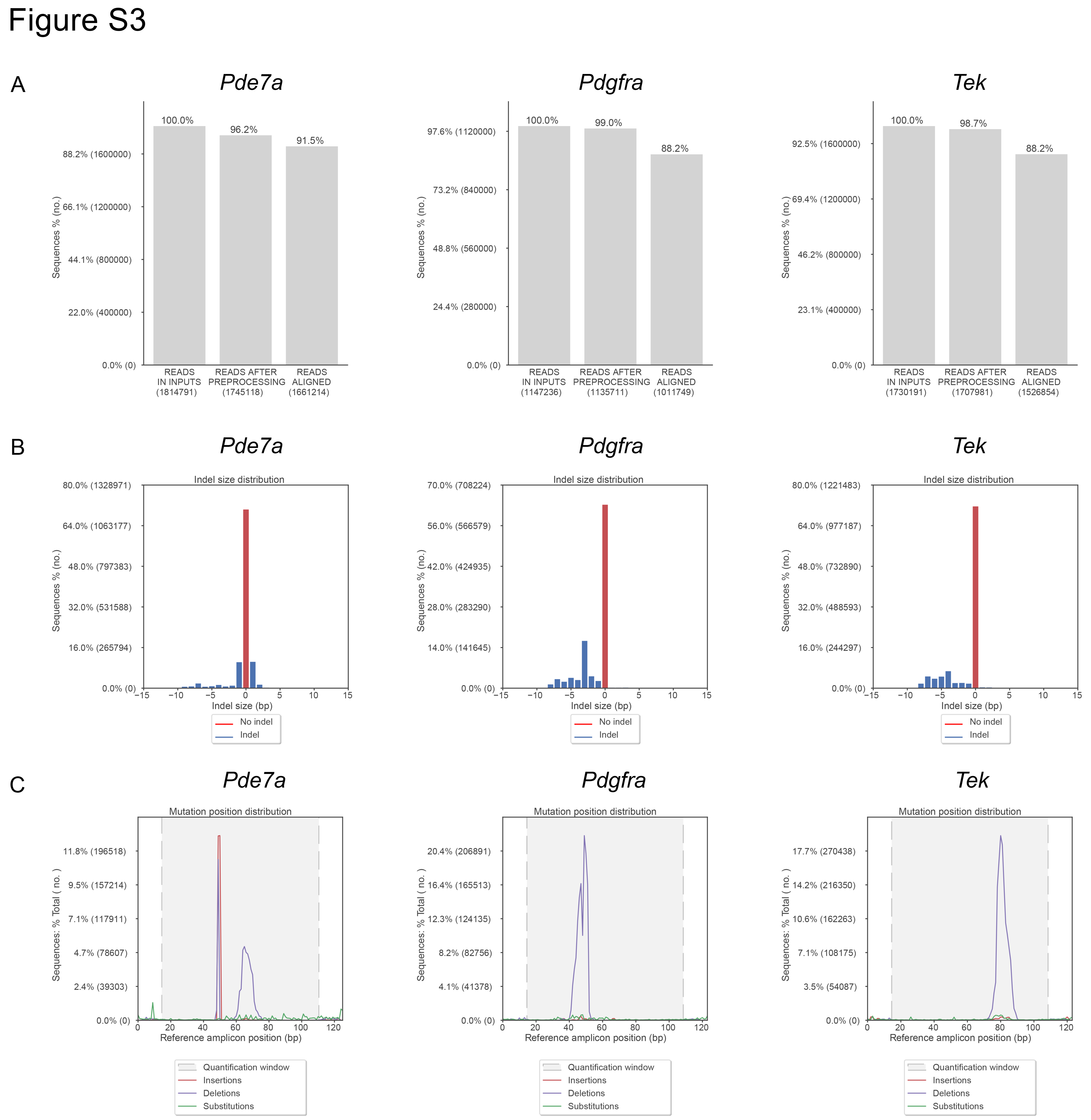

Supplement: Supplementary file 3 — Figure S3: Indels were induced by CeCas12a in Hepa1‐6 cells. (A) The number of reads in input fastqs after preprocessing and alignment with amplicons. (B) Frequency distribution of alleles with indels (blue) and without indels (red). (C) Frequency of insertions (red), deletions (purple) and substitutions (green) across the entire amplicon, including modifications outside of the quantification window. [file CTM2-14-e1758-s003.tif]

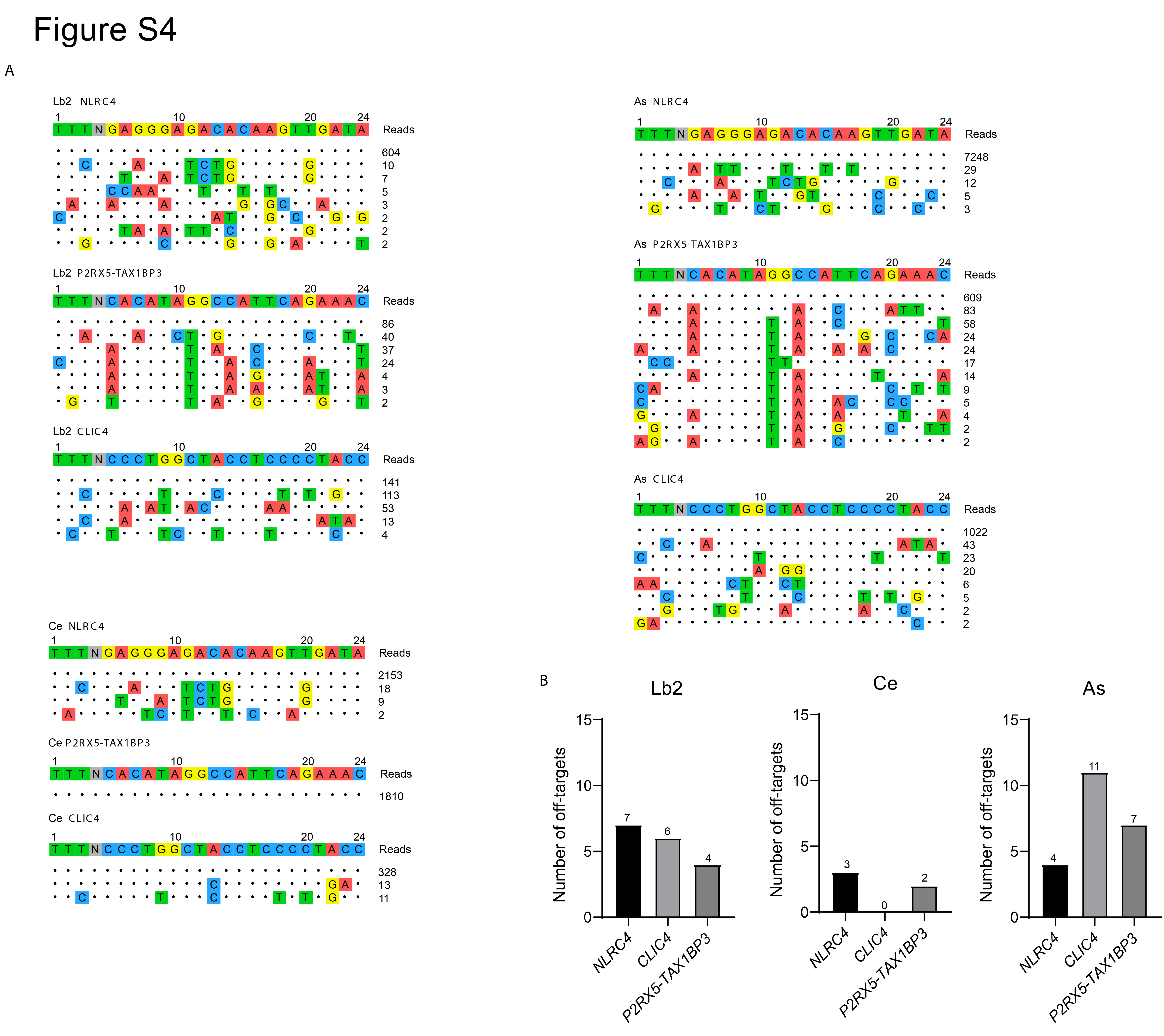

Supplement: Supplementary file 4 — Figure S4: Genome‐wide specificities of Cas12a orthologue‐matched crRNAs targeting three endogenous sites. (A) On‐targets and off‐target sites for AsCas12a, CeCas12a and Lb2Cas12a with crRNAs targeting three endogenous sites determined using GUIDE‐seq in 293T cells. (B) Summary of the detailed number of off‐target sites identified using GUIDE‐seq for AsCas12a, CeCas12a and Lb2Cas12a with crRNAs targeting three endogenous sites. [file CTM2-14-e1758-s001.tif]

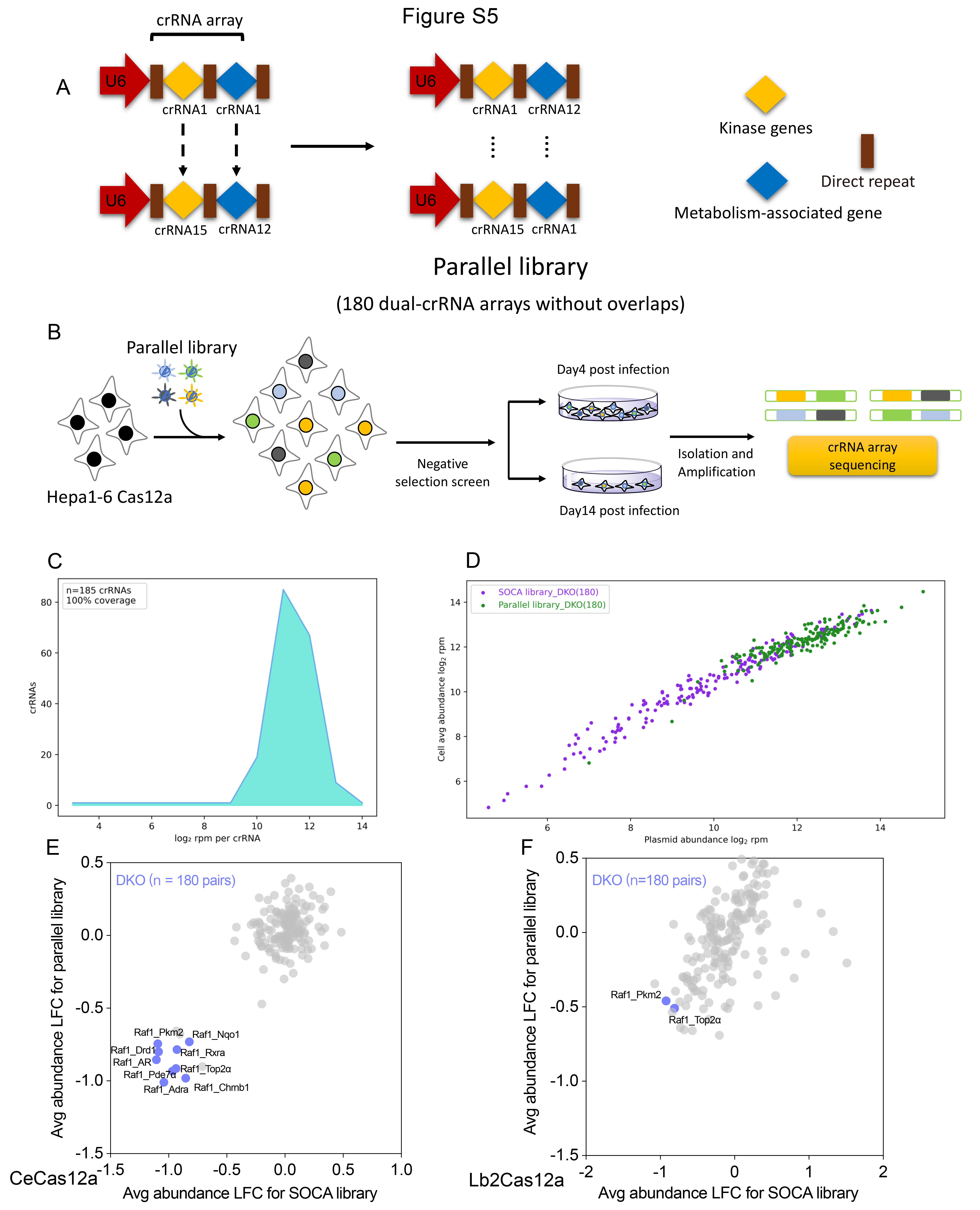

Supplement: Supplementary file 5 — Figure S5: Parallel library‐mediated double‐knockout screening confirmed the rationality of SOCA library construction. (A) Schematic describing the design for the parallel library. (B) Schematic of parallel library‐ mediated combinatorial screening in vitro. (C) A density plot depicting the abundance distribution of a parallel library. NGS sequencing determined 100% library coverage, consisting of 180 crRNA arrays. (D) Scatter plot depicting the abundance of the SOCA library and parallel library in the plasmid (n = 1) and averaged cell pools (n = 3). (E) The scatter plot compares the screening results for the parallel and SOCA libraries in CeCas12a‐positive cells. (F) The scatter plot compares the parallel and SOCA library screening results in Lb2Cas12a‐positive cells. [file CTM2-14-e1758-s005.tif]

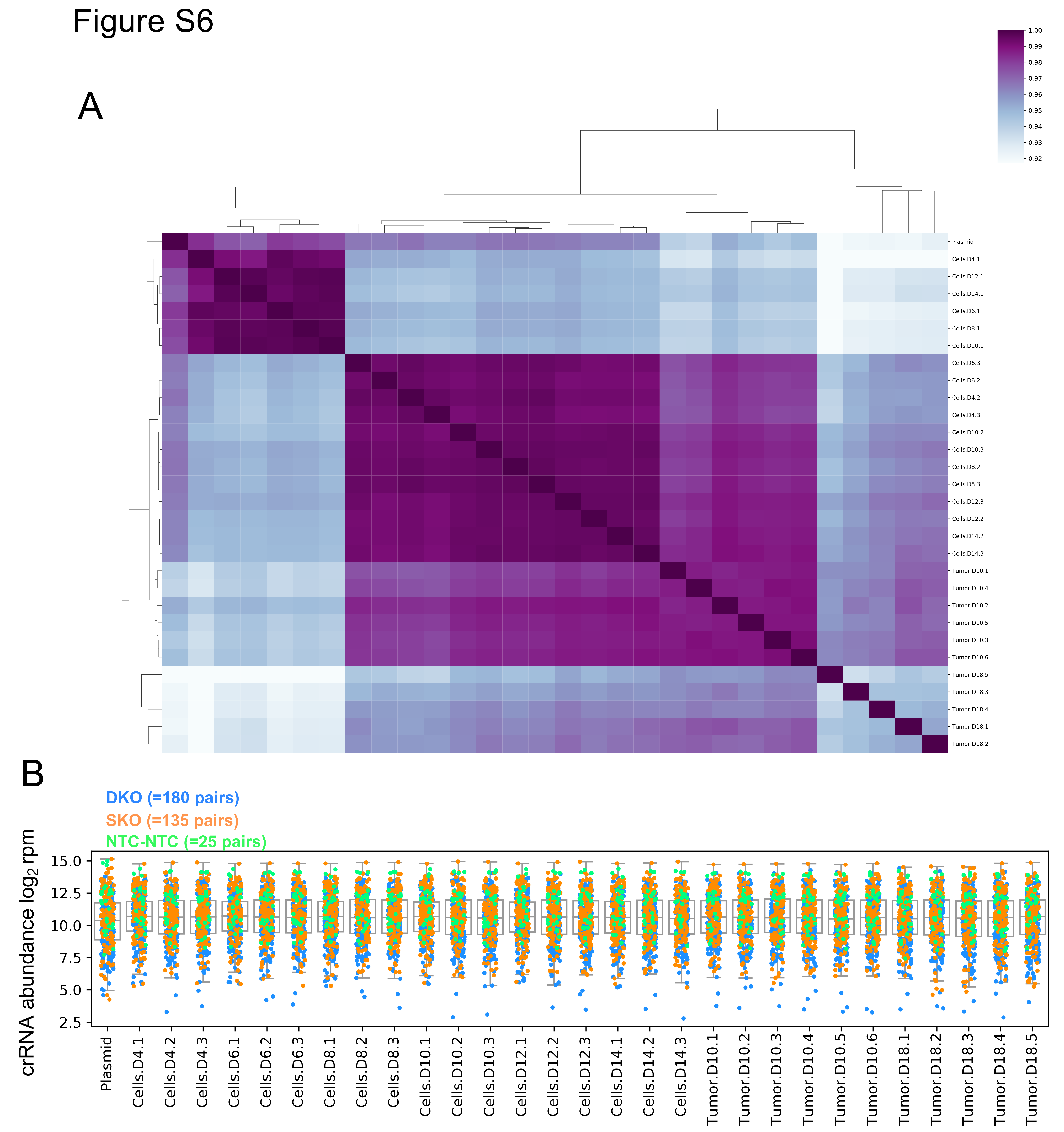

Supplement: Supplementary file 6 — Figure S6: Abundance analysis of the SOCA library in plasmid, cells and primary tumours. (A) Heatmap that shows the Spearman's correlation coefficients for crRNA array log2 rpm abundance between SOCA plasmid library (n = 1), SOCA library transduced cells (n = 3 cell replicates) and primary tumours (n = 5‒6 nu/nu mice). (B) Tukey's boxplots showing the distribution of crRNA array log2 rpm abundance between SOCA plasmid library (n = 1), SOCA library transduced cells (n = 3 cell replicates), and primary tumours (n = 5‒6 nu/nu mice). [file CTM2-14-e1758-s006.tif]

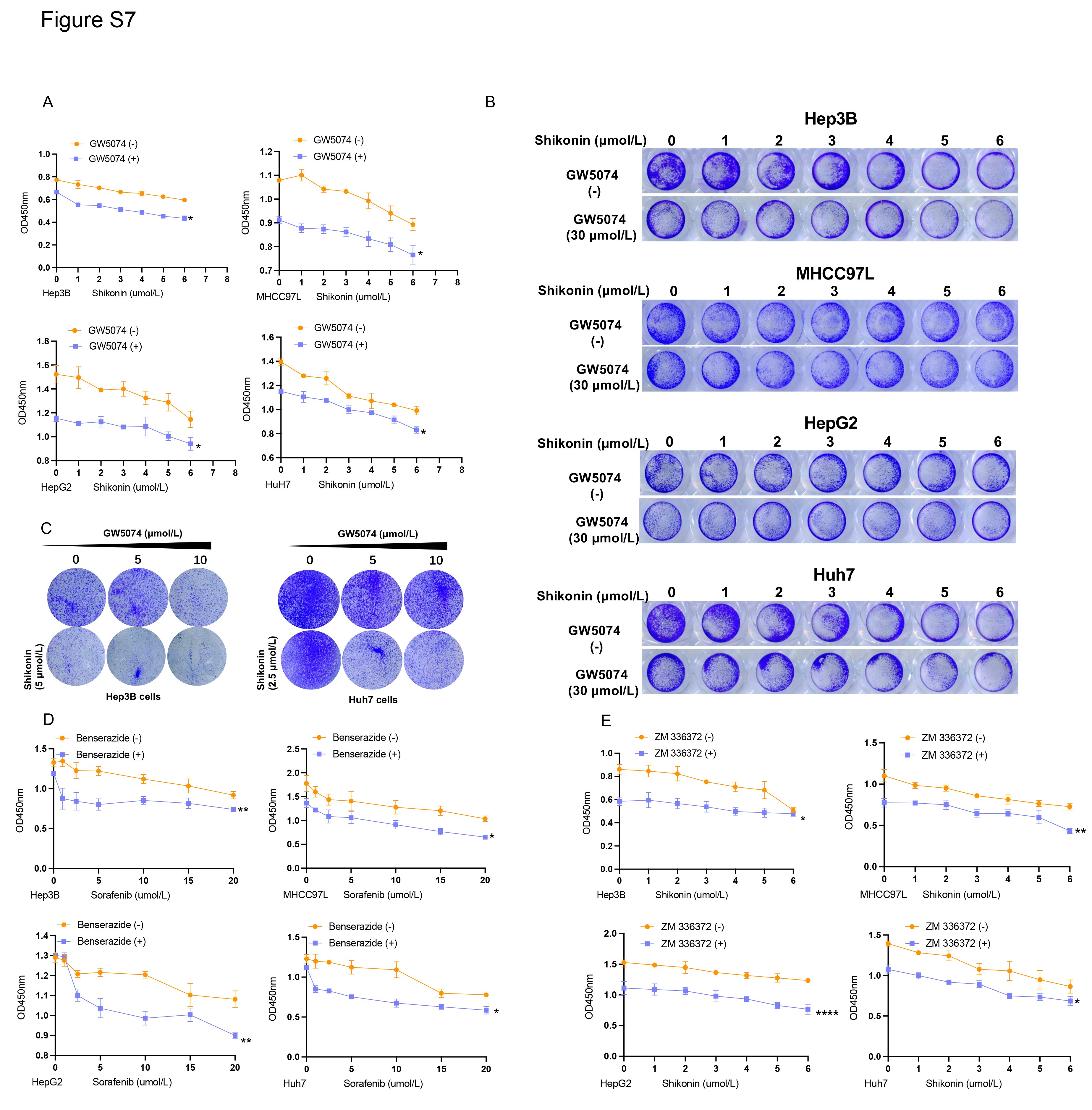

Supplement: Supplementary file 7 — Figure S7: Effect of specific compounds of Raf1 and Pkm2 on the activity of HCC cells. (A) Quantitative analysis of GW5074 in combination with shikonin after treatment of hepatocellular carcinoma cells in vitro. (B) Shikonin in combination with GW5074 inhibited the proliferation of Hep3B, MHCC97L, HepG2 and Huh7 cells. (C) Long‐term colony formation assays show that GW5074 combined with shikonin exhibits synergistic inhibition in Hep3B and Huh7 cells. (D) Quantitative analysis of benserazide in combination with sorafenib after treatment of hepatocellular carcinoma cells in vitro. (E) Quantitative analysis of ZM 336372 in combination with shikonin after treatment of hepatocellular carcinoma cells in vitro. [file CTM2-14-e1758-s008.tif]

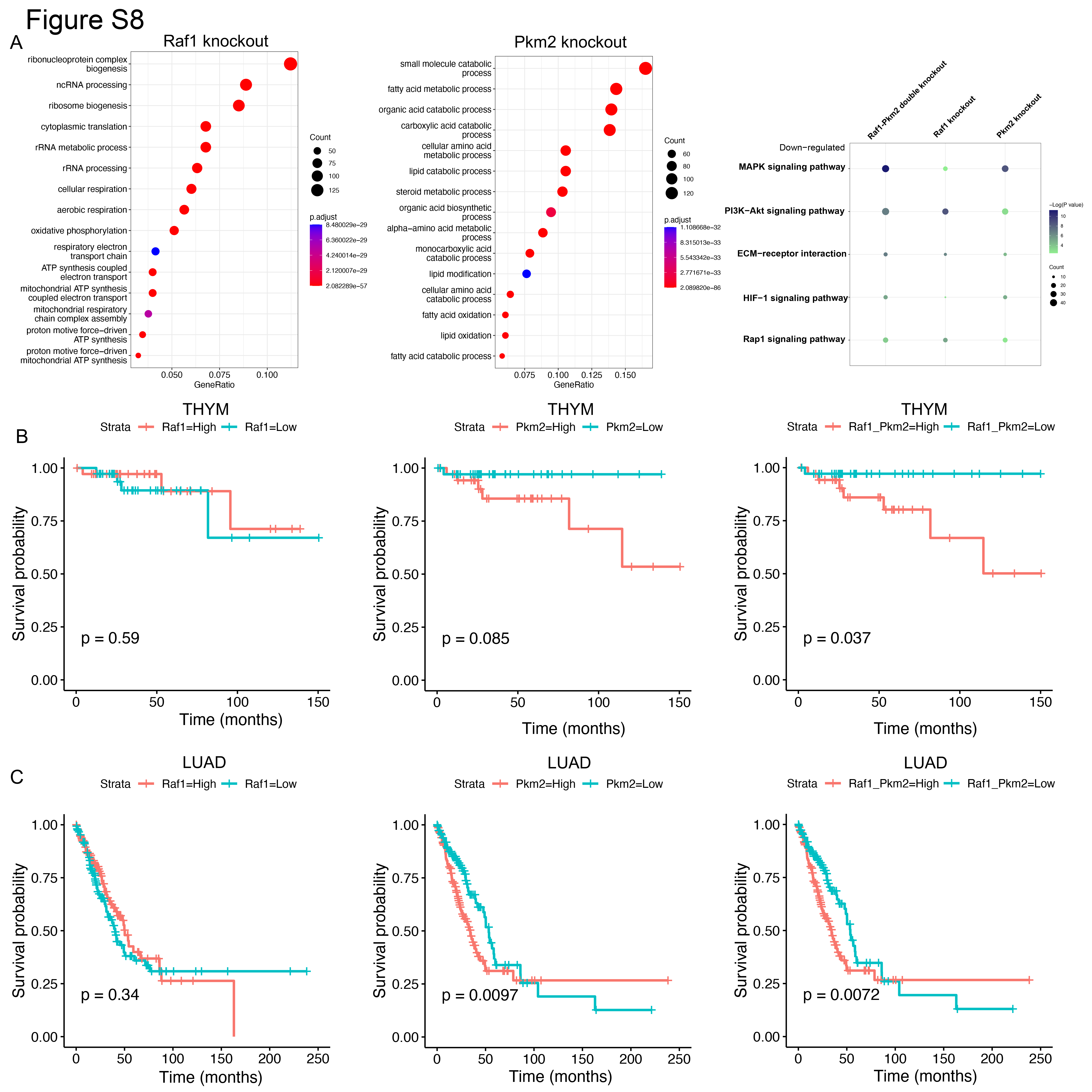

Supplement: Supplementary file 8 — Figure S8: The GO enrichment analysis for the single knockout of Raf1 and Pkm2 in the TCGA database and Kaplan–Meier curves depicting the effect of the Raf1_Pkm2 combination on overall survival for other cancers. (A) Pathway enrichment of genes negatively correlated with Raf1 expression and Pkm2 expression; representative KEGG pathways analysis showed that DEGs were enriched in downregulated gene sets. (B) Overall survival of patients with THYM under high or low expression for Raf1, Pkm and Raf1_Pkm2 from the TCGA database. (C) Overall survival of patients with LUAD under high or low expression for Raf1, Pkm and Raf1_Pkm2 from the TCGA database. THYM: thymoma; LUAD: lung adenocarcinoma. [file CTM2-14-e1758-s002.tif]

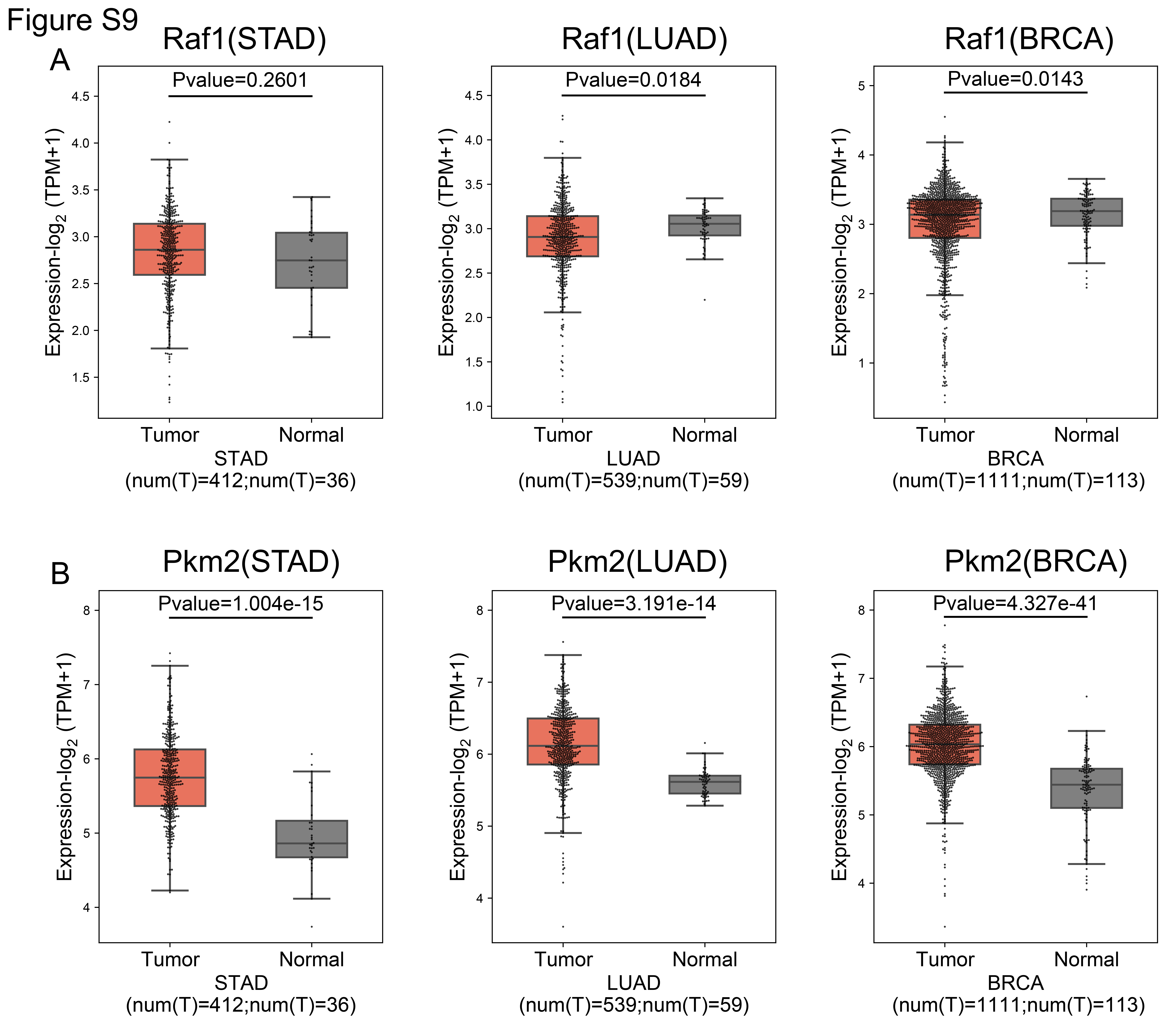

Supplement: Supplementary file 9 — Figure S9: Boxplots of Raf1 and Pkm2 expression in a variety of tumour samples from the TCGA database. (A) Boxplots of Raf1 expression in STAD, LUAD and BRCA samples. (B) Boxplots of Pkm2 expression in STAD, LUAD and BRCA samples. STAD: stomach adenocarcinoma; LUAD: lung adenocarcinoma; BRCA: breast cancer; num(T): number of tumour samples. [file CTM2-14-e1758-s009.tif]
